# Inter and intra-host diversity of RSV in hematopoietic stem cell transplant adults with normal and delayed viral clearance

**DOI:** 10.1093/ve/vead086

**Published:** 2023-12-28

**Authors:** Vasanthi Avadhanula, Daniel Paiva Agustinho, Vipin Kumar Menon, Roy F Chemaly, Dimpy P Shah, Xiang Qin, Anil Surathu, Harshavardhan Doddapaneni, Donna M Muzny, Ginger A Metcalf, Sara Javornik Cregeen, Richard A Gibbs, Joseph F Petrosino, Fritz J Sedlazeck, Pedro A Piedra

**Affiliations:** Human Genome Sequencing Center, Baylor College of Medicine, Houston, TX 77030, USA; Human Genome Sequencing Center, Baylor College of Medicine, Houston, TX 77030, USA; Department of Molecular and Human Genetics, Baylor College of Medicine, Houston, TX 77030, USA; Departments of Infectious Diseases, Infection Control & Employee Health, The University of Texas MD Anderson Cancer Center, Houston, TX 77030, USA; Department of Population Health Sciences, Mays Cancer Center, The University of Texas Health Science Center at San Antonio, San Antonio, TX 78229, USA; Human Genome Sequencing Center, Baylor College of Medicine, Houston, TX 77030, USA; Department of Molecular and Human Genetics, Baylor College of Medicine, Houston, TX 77030, USA; Department of Molecular Virology and Microbiology, Baylor College of Medicine, Houston, TX 77030,USA; Human Genome Sequencing Center, Baylor College of Medicine, Houston, TX 77030, USA; Department of Molecular and Human Genetics, Baylor College of Medicine, Houston, TX 77030, USA; Human Genome Sequencing Center, Baylor College of Medicine, Houston, TX 77030, USA; Department of Molecular and Human Genetics, Baylor College of Medicine, Houston, TX 77030, USA; Human Genome Sequencing Center, Baylor College of Medicine, Houston, TX 77030, USA; Department of Molecular and Human Genetics, Baylor College of Medicine, Houston, TX 77030, USA; Department of Molecular Virology and Microbiology, Baylor College of Medicine, Houston, TX 77030,USA; Human Genome Sequencing Center, Baylor College of Medicine, Houston, TX 77030, USA; Department of Molecular and Human Genetics, Baylor College of Medicine, Houston, TX 77030, USA; Department of Molecular Virology and Microbiology, Baylor College of Medicine, Houston, TX 77030,USA; Human Genome Sequencing Center, Baylor College of Medicine, Houston, TX 77030, USA; Department of Molecular and Human Genetics, Baylor College of Medicine, Houston, TX 77030, USA; Department of Computer Science, Rice University, Houston, TX 77030, USA; Department of Molecular Virology and Microbiology, Baylor College of Medicine, Houston, TX 77030,USA; Department of Molecular Virology and Microbiology, Baylor College of Medicine, Houston, TX 77030,USA; Department of Pediatrics, Baylor College of Medicine, Houston, TX 77030, USA

**Keywords:** RSV, variants, intra-host, inter-host, hematopoietic stem cell transplant adults, HCT

## Abstract

Respiratory syncytial virus (RSV) infection in immunocompromised individuals often leads to prolonged illness, progression to severe lower respiratory tract infection, and even death. How the host immune environment of the hematopoietic stem cell transplant (HCT) adults can affect viral genetic variation during an acute infection is not understood well. In the present study, we performed whole genome sequencing of RSV/A or RSV/B from samples collected longitudinally from HCT adults with normal (<14 days) and delayed (≥14 days) RSV clearance who were enrolled in a ribavirin trial. We determined the inter-host and intra-host genetic variation of RSV and the effect of mutations on putative glycosylation sites. The inter-host variation of RSV is centered in the attachment (G) and fusion (F) glycoprotein genes followed by polymerase (L) and matrix (M) genes. Interestingly, the overall genetic variation was constant between normal and delayed clearance groups for both RSV/A and RSV/B. Intra-host variation primarily occurred in the G gene followed by non-structural protein (NS1) and L genes; however, gain or loss of stop codons and frameshift mutations appeared only in the G gene and only in the delayed viral clearance group. Potential gain or loss of O-linked glycosylation sites in the G gene occurred both in RSV/A and RSV/B isolates. For RSV F gene, loss of N-linked glycosylation site occurred in three RSV/B isolates within an antigenic epitope. Both oral and aerosolized ribavirin did not cause any mutations in the L gene. In summary, prolonged viral shedding and immune deficiency resulted in RSV variation, especially in structural mutations in the G gene, possibly associated with immune evasion. Therefore, sequencing and monitoring of RSV isolates from immunocompromised patients are crucial as they can create escape mutants that can impact the effectiveness of upcoming vaccines and treatments.

## Introduction

Respiratory syncytial virus (RSV) is the leading cause of lower respiratory tract infections (LRTI) in children worldwide, which also contributes to significant morbidity and mortality in elderly adults and immunocompromised individuals (Falsey et al. 2005; Nair et al. 2010; Shi et al. 2017). In older adults, RSV causes ∼ 200,000 hospitalization and 10,000–14,000 deaths in the United States (McLaughlin et al. 2022). In immunocompromised individuals, RSV infections can quickly progress from upper to lower respiratory tract in up to 50 per cent of the individuals, and 24–30 per cent of RSV LRTI may result in death (Whimbey et al. 1995; Waghmare et al. 2017; Chatzis et al. 2018). In hematopoietic stem cell transplant (HCT) patients, lymphopenia, allogeneic transplant, graft-versus-host disease, and diagnosis of infection within one month after transplantation have been associated with life-threatening RSV disease (Kim et al. 2014), (Shah et al. 2012).

In addition to host factors, viral loads can play an important role in the progression of RSV disease. In infants, high RSV viral loads early in infection are linked with less severe disease, possibly due to rapid and robust engagement of the innate immune responses (Passariello et al. 2006; Piedra et al. 2017; Thwaites et al. 2018). Meanwhile, high viral loads later in infection have been associated with greater disease severity, especially in hospitalized patients (Hasegawa et al. 2015). Inability to control viral replication can determine the duration of illness. Prolonged shedding for >4 weeks has been documented in hospitalized infants. HCT patients with an impaired cellular immunity are at risk of extended viral shedding and high mortality (Renaud and Englund 2016). Although viral load and duration of RSV shedding play an important role in disease outcome and transmission, a less understood dynamic is the inter-host and intra-host genetic diversity of the virus over time and under varying immune pressures.

RSV has a negative-sense, single-stranded, non-segmented genome with 15,200 nucleotides that encodes for eleven proteins. The attachment (G) and fusion (F) glycoproteins are the main target for the host’s humoral and mucosal antibody responses (Collins and Melero 2011). RSV is divided into two antigenic subtypes (RSV/A and RSV/B) largely driven by the nucleotide and antigenic differences in the G glycoprotein (Anderson et al. 1985). RSV/A and RSV/B genotypes emerged and are replaced periodically. The most recent genotypes circulating worldwide are RSV/A/Ontario (ON) and RSV/B/Buenos Aires (BA) (Trento et al. 2010; Avadhanula et al. 2015; Duvvuri et al. 2015). RSV undergoes dynamic evolutionary process, especially in the G and F genes, to evade the host’s humoral and mucosal immunity (Yu et al. 2021), (Tan et al. 2013). This mechanism of immune evasion can result in slow antigenic drift and give rise to treatment-emergent variants that can impact the efficacy of monoclonal antibodies or antiviral therapies targeting the relatively conserved F protein or the polymerase (L) proteins (Zhu et al. 2011; Yan et al. 2014; Deval et al. 2016; Simões et al. 2021).

How inter-host and intra-host variations impact molecular evolution and thereby RSV disease severity is unclear. There is a lack of understanding of how RSV variation occurs during the presence or absence of adaptive immune pressure and longitudinally in immunocompromised adults. We previously reported on a cohort of naturally RSV-infected HCT recipients that were enrolled in a randomized controlled trial of ribavirin. Approximately, 50 per cent of these adults cleared RSV within 2 weeks of virus identification (normal viral clearance), and the other half experienced delayed viral clearance. Normal clearance of RSV correlated with significant rises in both serum-neutralizing antibody titers and palivizumab-like antibody concentration (Avadhanula et al. 2015), (Ye et al. 2018), (Ye et al. 2021). The RSV-infected HCT adults were randomized to receive either oral or aerosolized ribavirin or served as no treatment control because of their immunodeficiency scoring index. In the present study, we determined the viral genetic diversity on samples collected over time in HCT subjects infected with the current genotypes, RSV/A/ON, or RSV/B/BA. We hypothesized that greater variation would occur in individuals with delayed viral clearance because of their inability to efficiently control virus replication in a reduced immune pressure environment. Thus, we monitored the genomic changes and investigated the role of viral variants on protein function, antigenicity, and glycosylation patterns in HCT adults with normal and delayed RSV clearance.

## Results

### Study cohort and phylogenetic analysis

This 4-year ribavirin treatment trial enrolled forty adult HCT recipients infected with RSV; twenty-two were infected with RSV/A and eighteen with RSV/B. In addition, the cohort was stratified by duration of RSV shedding: normal viral clearance (<14 days) and delayed viral clearance (≥14 days). The demographics of the cohort stratified by RSV subtype or duration of RSV shedding have been previously reported. The only statistically significant difference observed was that the delayed viral clearance group was more likely to receive an allogenic cell transplant compared to normal viral clearance group (18/20 versus 11/20, *P* <0.025) and HCT recipients, where normal viral clearance generated significantly higher neutralizing and RSV F site-specific competitive antibodies(Avadhanula et al. 2015), (Ye et al. 2018), (Ye et al. 2019), (Ye et al. 2020). This cohort provided us an opportunity to evaluate inter- and intra-host genetic diversity, potential changes in glycosylation patterns based on infecting subtype and viral clearance status, and the effect of antiviral treatment.

**Figure 1.**
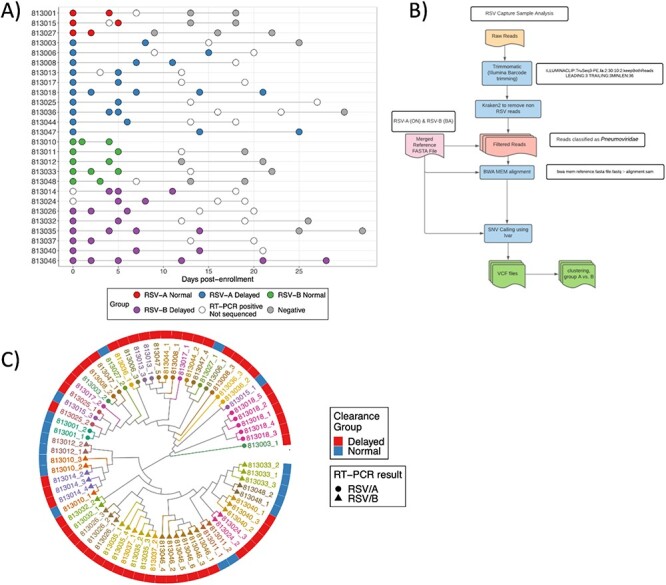
A) The distribution of subjects in the cohort according to subtype (RSV/A or RSV/B) and clearance time (normal or delayed clearance). Each circle represents a sample collection timepoint. Filled circles are samples with full-length sequence, empty circles are samples that could not be sequenced, and gray circle are samples that tested negative for RSV. The *x*-axis represents the day post-enrollment at each visit. B) The analysis pipeline. RSV was sequenced using RSV-specific capture probes enrichment method. After sequencing, reads were filtered for the Pneumoviridae family using Kraken2 and aligned to a concatenated RSV/A and RSV/B reference genomes (ON and BA, respectively), reads that aligned to respective RSV subtype as defined by PCR were kept. Single nucleotide variants and indels were then called using iVar. C) Phylogenetic tree of RSV/A and RSV/B isolates. Samples from the same subject are represented by unique color, and labels represent patient and their visit number separated by an underscore. The heatmap external to the tree represents viral clearance groups.

Nasal wash samples collected prospectively during the first 4 weeks after enrollments were sequenced. The sequencing analysis was performed on a subset of the original cohort; twenty-six of forty HCT subjects generated complete viral genomes. These subjects were divided based on their infecting RSV subtype and by viral clearance. The number of subjects, their longitudinal samples collected over time, and their sequencing status are depicted in Fig. 1A. Viral load by Ct value and viral shedding for each subject in normal and delayed viral clearance groups can be seen in Supplementary Fig. S1. The mean Ct values of RSV/A normal clearance group was 24.1 ± 1.56 and delayed clearance group was 20.76 ± 3, while for RSV/B, the average Ct values were 23 ± 3.05 for normal clearance and 21.55 ± 5 for delayed clearance. There was statistical difference in Ct values between normal and delayed clearance groups for RSV/A (*P* = 0.03, Student’s t test) while there was no statistical difference in Ct value between normal and delayed clearance groups for RSV/B. The sequencing analysis pipeline and variant calling are displayed in the methods and Fig. 1B. Variants were filtered by a minimum coverage depth of 30*×* and minimal allele frequency of 20 per cent to avoid sequencing artifacts or preparation errors. Only variants that mapped to the RSV subtype as determined by PCR were kept. Phylogenetic analysis was performed using read2tree (Dylus et al. 2023) and as expected, almost all samples originating from the same patient clustered closely together (Fig. 1C).

### Inter-host genetic diversity of RSV/A and RSV/B by clearance group

**Figure 2.**
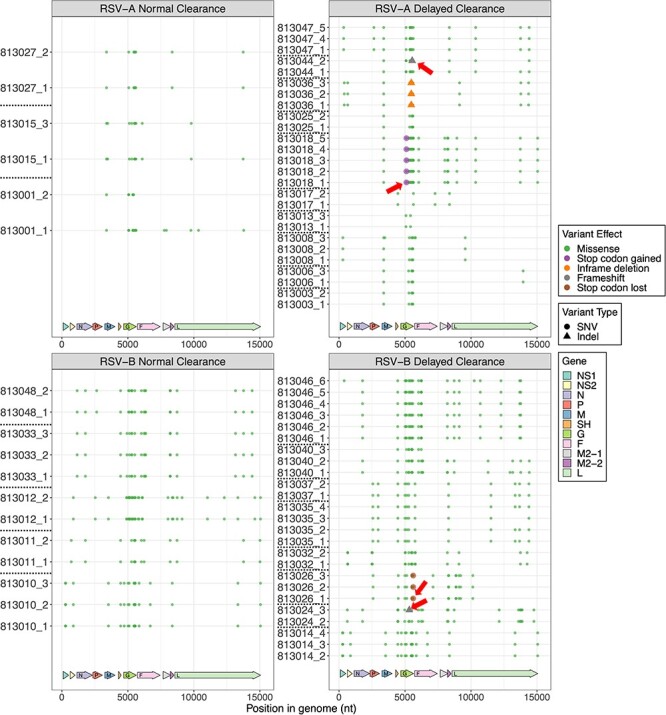
Representation of all non-synonymous and non-UTR variants among all samples. The four groups representing samples from both subtypes (RSV/A and RSV/B) and both clearance groups (normal or delayed clearance) are represented in individual plots. Different colors represent different variant effects, while different symbols represent single nucleotide variants or Indels. Only genomic differences that had a minimal 20 per cent allele frequency and 30× coverage were accepted as variants and are shown in this plot. All samples are represented as the patient with visit number separated by an underscore on *y*-axis. The *x*-axis represents the position in the genome, with the arrows in the bottom representing each gene position.

Using the analysis pipeline described above, we discovered a total of 461 different variants in RSV/A/ON isolates: 152 variants present in the normal group (three individuals, six total samples) and 356 in the delayed group (ten individuals, twenty-six total samples). RSV/B/BA isolates had a total of 762 variants: 373 in the normal group (five individuals, twelve total samples) and 623 in the delayed group (eight individuals, twenty-five total samples). These variants include single-nucleotide variants (SNVs) and structural variants, such as small insertion or deletions (indels; less than fifty nucleotides long). Figure 2 shows all non-synonymous variants (excluding variants in untranslated regions—UTR—and synonymous amino acid substitutions), while Supplementary Fig. S2 shows all variants. A complete list of all the variants and their effects can be seen in Supplementary Table S1. Many of the variants were already widespread among the isolates sequenced. Investigating the most widespread variants in our cohort, we observed eighty variants were common between five different subjects, eleven of those variants were found in RSV/A isolates, and sixty-nine in RSV/B. Similarly, there were seven variants that were common in at least ten different subjects, one in RSV/A and six in RSV/B (Supplementary Table S1). The apparent higher number of variants in RSV/B as compared to RSV/A are likely due to the reference sequence used to compare the isolates, where the consensus reference sequence for RSV/B was generated using isolates over a broader range in time (20 years) versus RSV/A (4 years) (see Methods), thereby showing greater divergence from the reference.

Comparison of the inter-host variation for normal and delayed groups for RSV/A showed that only one indel was present in one of three subjects in the normal clearance group whereas nine indels were present in eight of ten subjects in the delayed clearance group (0.3/subject for normal vs 0.9/subject for delayed) (Supplementary Fig. S2 and Table 1). Comparing missense mutations, a total of twenty-nine unique missense mutations were present in three subjects in normal clearance, whereas sixty unique missense mutations were present in ten subjects in the delayed clearance group (9.6 missense mutations/subject for normal vs 6 missense mutations/subject for delayed clearance). Most of the missense variants accumulated in the G and L genes for both normal and delayed clearance. In addition, M gene in the normal and NS2 in delayed clearance groups had distinct changes (Fig. 2, Supplementary Fig. S2, and Table 1). Interestingly, for RSV/A normal clearance groups, 83 per cent of the variants in G gene appeared only in one subject at one time, and 16 per cent occurred in two subjects simultaneously, whereas for delayed group, 69 per cent appeared in one subject at one time, 18 per cent in two subjects, 9 per cent of mutants occurred in six subjects simultaneously. All these mutations were present in mucin-like regions in G gene (Supplementary Table S1).

**Table 1.** The average number of missense variants per gene for each RSV-subtype by clearance group.

Gene	RSV/A normal clearance	RSV/A-delayed clearance	RSV/B normal clearance	RSV/B-delayed clearance
NS1	0.00	0.30	0.40	0.38
NS2	0.00	0.10	0.60	0.50
N	0.00	0.00	1.00	0.38
P	0.00	0.10	0.40	0.88
M	1.33	0.90	0.40	0.13
SH	0.00	0.10	0.80	1.00
G	7.00	5.50	8.80	5.75
F	0.33	0.40	3.20	2.25
M2-1	0.67	0.20	1.20	0.63
M2-2	0.33	0.40	0.60	0.75
L	2.00	1.50	4.00	4.88

Comparison of the inter-host variation for normal and delayed groups for RSV/B showed that two indels were present in two of five subjects in the normal clearance group and five indels were present in six of eight subjects in the delayed clearance group (0.4/subject for normal vs 0.6/subject for delayed) (Supplementary Fig. S2 and Table 1). A total of seventy-three unique missense mutations were present in all five subjects of the normal clearance group, whereas 102 unique missense mutations were present in all eight subjects in the delayed clearance group (14.6/subject for normal vs 12.75/subject for delayed clearance group). Most of the missense variants accumulated in the G, F, and L genes for both normal and delayed clearance. However, isolates of the delayed clearance had distinct changes in P, SH, and L compared to the normal clearance group (Fig. 2, Supplementary Fig. S2, and Table 1). For the normal clearance group, 80 per cent of the variants in G gene appeared only in one subject at one time, and 14 per cent occurred in two subjects simultaneously, whereas for the delayed clearance group, 85 per cent appeared in one subject at one time, 6 per cent in two subjects, and 3 per cent of mutants each occurred in three, four, or six subjects concurrently. All these mutations were present in mucin-like regions or heparin binding domains in the G gene (Supplementary Table S1).

**Figure 3.**
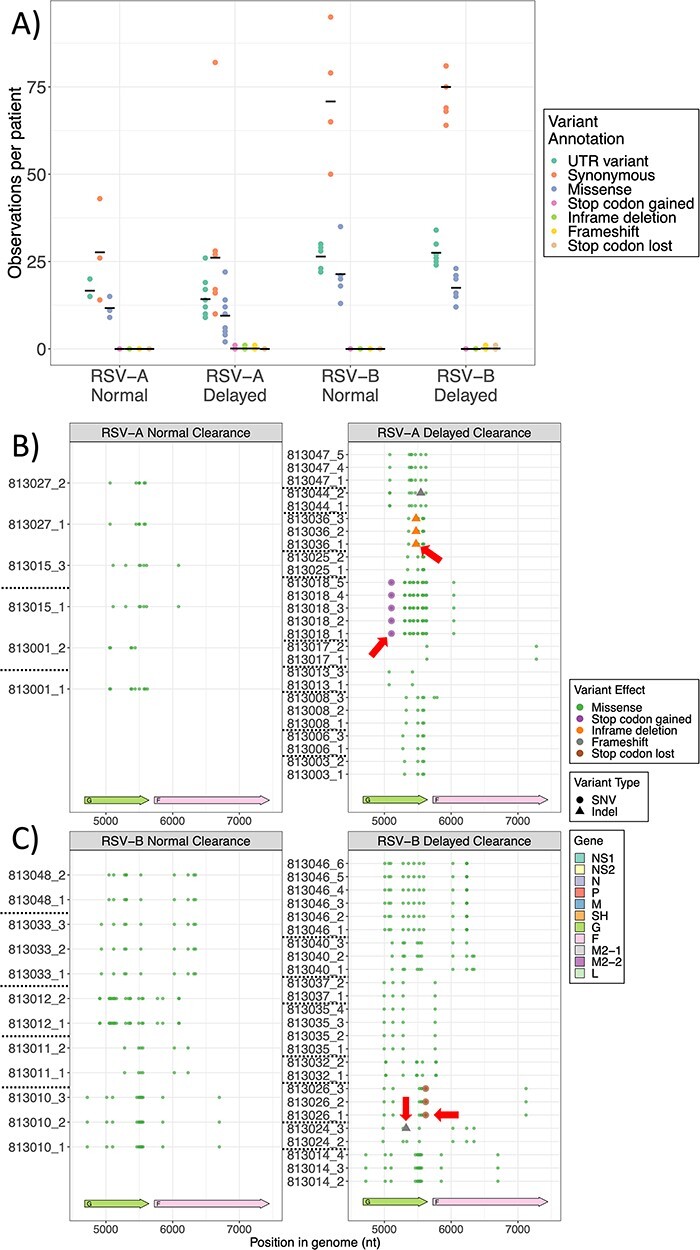
A) Number of variants per patient by RSV subtype and clearance status. Each point on the plot represents the count of a particular variant annotation (indicated by color) for an individual patient by RSV subtype (A or B) and clearance group (normal or delayed). Only genomic differences that had a minimal 20 per cent allele frequency and 30× coverage were accepted as variants and are shown in this plot. Black dashes represent the average number of observations for each variant type in each group. We employed One-way ANOVA with Tukey’s post-test to verify group differences. The number of the non-synonymous variants in the G and F genes per patient by RSV subtype clearance status is shown for B) RSV-A and C) RSV-B. Colors represent different variant annotations and shapes the variant type (single nucleotide variant (SNV), or indel). Red arrows indicate variants of interest (see text).

We next wanted to check if the mean number of variants per patient by RSV subtype was changed by the clearance group. There were no statistical differences in the mean number of variants per person by RSV subtypes between the normal and delayed clearance group for synonymous variants, missense variants, or variants in the UTR (Fig. 3A). Even analyzing each gene individually, there was no statistical difference between the clearance groups by RSV subtypes (Supplementary Fig. S3). We performed a correlation between the Ct value, a surrogate for viral copy number, and the number of variants observed during the first visit. Three of the four groups showed no statistical impact. For the RSV/B-delayed group, however, a significant correlation of Ct value and number of variants occurred (*R* = −0.9 and *P* = 0.004). Specifically, for this group, as measured by Ct values, the total number of variants exhibited a moderate inverse relationship (hence a positive correlation with viral copy number). Nevertheless, this correlation, while statistically meaningful, suggested only mild influences on the number of variants (Supplementary Fig. S4).

It was notable, however, most missense variants accumulated in the distal hypervariable region of the G gene for both RSV subtypes (Fig. 3B and C). Interestingly, the presence of variants with a potentially more deleterious role on viral biology, such as gain or loss of stop codons and frame shifts, was only observed in the RSV/A and RSV/B groups with delayed viral clearance (Fig. 3B and C), even though they only appeared in a few subjects. For instance, patient 813,018 from the RSV/A-delayed clearance group had a variant resulting in a new stop codon in gene G, which would result in a shorter G protein containing just 142 instead of 321 amino acids (Fig. 3B**.—**red arrow). While patient 813,026 (RSV/B-delayed clearance) displayed a loss of the G gene stop codon, suggesting the formation of chimeric G-F protein (Fig. 3C). Interestingly, in one RSV/A patient (patient 813,015) in our cohort, we observed T122A mutation in the F gene, which has been seen in RSV strains from recent 2022–23 outbreak (Goya et al. 2023). We also observed two frameshift variants in gene G in samples from two distinct subjects within the delayed clearance groups, where one patient infected with RSV/A (patient 813,044; Fig. 3B—red arrow) and one with RSV/B (patient 813,024, Fig. 3C—red arrow). These variants appeared at the second and third visits, respectively, and were at position 289 and 216 on the G protein of RSV/A and B, which can potentially have an implication on the structure and function of the G protein. We observed only three missense variants in the M gene of RSV/A isolates, one of which (I43M) was found in eleven subjects. The other two mutations were present in two (M73L) or one (V72I) patient. Only one M protein variant was found in RSV/B (T89I in three subjects). RSV/A isolates presented only three different missense variants in NS1 and one in NS2 genes, and all four variants were present in a single patient only. RSV/B isolates showed three missense variants in NS1 (two of them present in two different subjects and the third in a single patient) and five in NS2 (one present in three distinct subjects and the rest in a single patient). Further details can be found in Supplementary Tables S1 and S2.

#### Intra-host diversity of RSV/A and RSV/B by clearance groups

**Figure 4.**
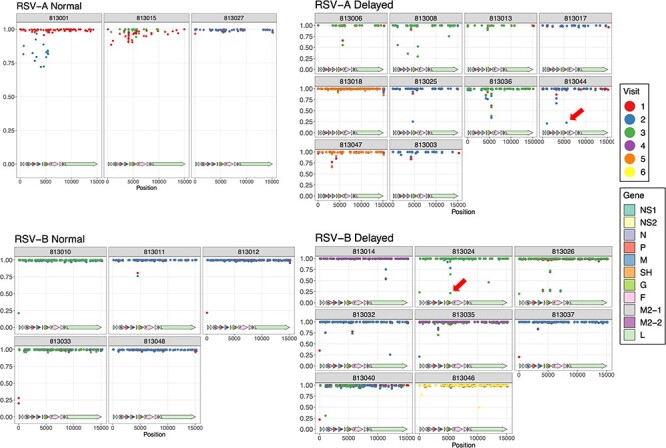
Allele frequencies for all variants across the whole genome. Each panel represents an individual subject, and each color represents a different visit for that subject. Only genomic differences that had a minimal 20 per cent allele frequency and 30× coverage were accepted as variants and are shown in this plot. Panels were grouped according to RSV subtype and clearance group. The *y*-axis represents the allele frequency, and the *x*-axis represents the genomic position.

We next wanted to evaluate the intra-host diversity by measuring allele frequency for each variant by patient, infecting RSV subtype and viral clearance status (Fig. 4). Variants with frequencies between 0 and 100 per cent indicate that both alleles are present at different ratios in the viral population. Most of the variants occurred at a 100 per cent frequency at enrollment. In subsequent visits, some subjects developed varying frequencies of mixed variant populations. In RSV/A infected HCT adults, two of the three subjects (813,001 and 813,015) in the normal clearance group had variable allele frequencies for multiple variants. Subject 813,001 displayed mixed variant populations in the N, P, M, and G genes in the second visit, while subject 813,015 had mixed variant populations in first visit that stabilized at visit 3 (Fig. 4). In contrast, six of ten subjects in RSV/A delayed clearance group had mixed variant populations detected at later visits that clustered in the G gene. For RSV/B adults with normal viral clearance, four of five developed a stabilized variant population after the first visit, while seven of eight individuals in the RSV/B-delayed clearance group developed mixed population variants in subsequent visits.

**Figure 5.**
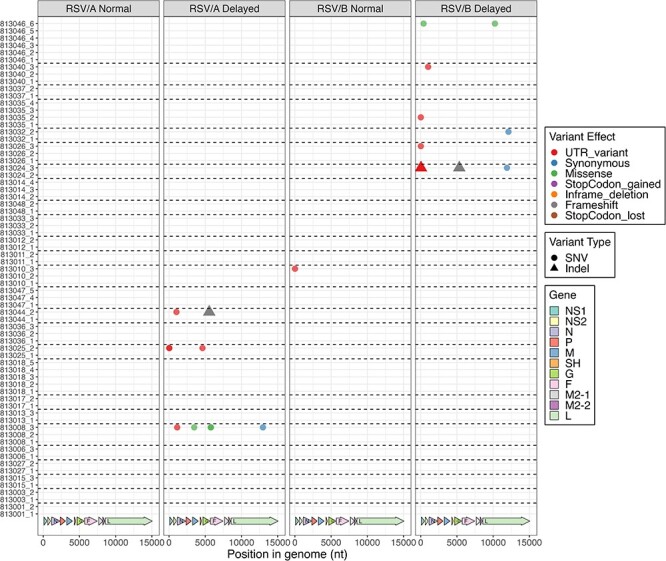
Representation of new intra-host variants appearing after visit 1 for each subject. The four groups representing samples from both subtypes (RSV/A and RSV/B) and both clearance groups (normal or late clearance) are represented in individual plots. Only variants considered to be new after the first visit are represented. Different colors represent different variant effects, while different symbols represent SNVs or indels. Only genomic differences that had a minimal 20 per cent allele frequency and 30*×* coverage were accepted as variants and are shown in this plot. All samples are represented as the patient number and visit number separated by an underscore on *y*-axis. The *x*-axis represents the position in the RSV genome, with the arrows representing each gene position.

The variants observed in the delayed clearance group involved the gain or loss of stop codons in the G gene in subjects 813,018 (RSV/A) and 813,025 (RSV/B), respectively, and were all at 100 per cent allele frequency and remained stable throughout all visits (Fig. 3, red arrows, and Fig. 4). Frameshift variants occurring in the G gene in subjects 813,044 (RSV/A) and 813,024 (RSV/B) were observed only once for each subject, and both were detected only after their first visit (Fig. 5). Their allele frequencies were ∼23 per cent and ∼22 per cent, respectively (Fig. 4, red arrows), which were just above our minimum allele frequency cut-off of 20 per cent. Interestingly, subject 813,044 at enrollment did not show evidence of existence of the allele, suggesting that this variant appeared *de novo* between enrollment (first visit) and the second visit. On the other hand, the frameshift variant that occurred on third visit in subject 813,024 was already present at enrollment at ∼9 per cent, which was below our cutoff of 20 per cent.

We next evaluated the emergence of new intra-host variants after the first visit, whereby the sequence from first visit of each subject served as a reference for sequences from subsequent visits. Although we did not reach statistical significance (*P* = 0.19, Fisher exact test) on the number of new intra-host variants after first visit, between normal and delayed groups (see Methods), we found that none of the subjects in normal groups for RSV/A and only one subject (813,010) in the RSV/B normal viral clearance group displayed any new intra-host variants. Subject 813,010 for RSV/B had one in-frame deletion at 5ʹUTR of NS1 at visit 3 (Fig. 5 and Supplementary Table S1). However, both RSV/A and RSV/B-delayed viral clearance groups showed new intra-host variants after the initial visit. For RSV/A, subject 813,008 developed an UTR-variant of N, missense mutations in M (V72I) and F (T8S, C21Y) at second visit, subject 813,025 developed an UTR-variant of NS1 and G, and subject 813,044 had a UTR-variant of N and frameshift in G (L289fs—Fig. 3 red arrow and Fig. 5) at the third visit. For RSV/B, subject 813,024 had mutation in UTR of NS1 and a frameshift in G (T216fs) in visit 3 (Fig. 3 red arrow and Fig. 5). Subjects 813,026 and 8,133,035 had mutations in the UTR of NS1, subject 813,040 had a mutation in the UTR of N whereas subject 813,046 developed missense mutations in NS1(C98Y) and L (N564D) (Fig. 5 and Supplementary Table S1).

#### Potential changes in protein glycosylation

**Figure 6.**
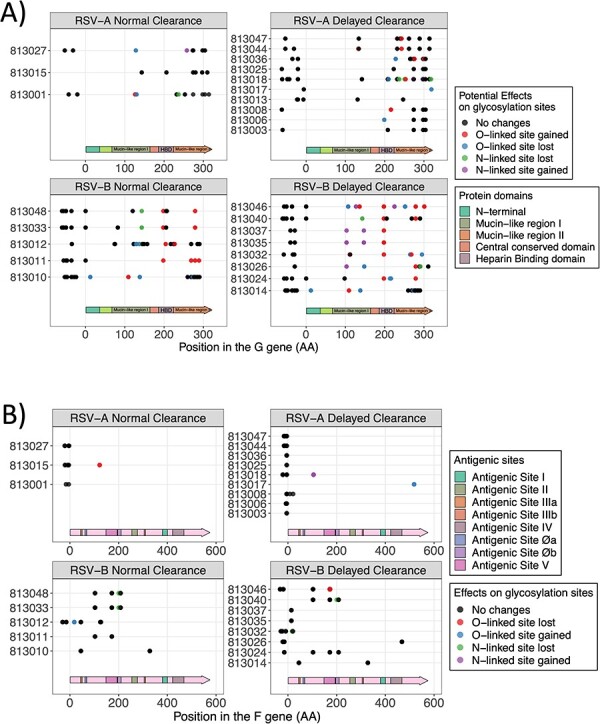
Potential glycosylation changes in proteins G and F. All non-synonymous variants present in the first visits of each patient are represented here. Any gain or loss of an asparagine due to a missense variant is categorized as a potential N-linked glycosylation site gain or loss, respectively. Likewise, gain or loss of either a serine or a threonine is categorized as a potential gain or loss of an O-linked glycosylation site, respectively. Any other changes that do not involve these amino acids are categorized as ‘no changes’. Only genomic differences that had a minimal 20 per cent allele frequency and 30*×* coverage were accepted as variants and are shown in this plot. A) Variants with potential changes in glycosylation sites in the G protein domains. B) Variants with potential changes in glycosylation sites in the F protein domains.

N-linked and O-linked glycosylation are important post-translational modifications, particularly in the G and F proteins in RSV. The G protein is known to contain an abundance of N- and O-linked glycans while the F protein contains five or six N-linked glycans only. Changes in glycosylation can directly affect the protein’s folding and stability, and hence its function in antigenicity and immune evasion (Cane 1997). N-linked glycosylation occurs on Asparagine residues, while O-linked glycosylation occurs on Serine or Threonine residues (Johnson et al. 1987). We identified potential loss or gain of glycosylation sites by detecting substitutions for these amino acids on the G (Fig. 6A) and F (Fig. 6B) genes for the first initial visits of each subject.

Almost all changes in potential glycosylation sites for RSV/A and B subtypes at first initial visit were found in the proximal and distal third of the G gene, which encodes f or mucin-rich regions that contain high number of serine and threonine residues (potential O-linked glycosylation sites). Two of three subjects for the RSV/A normal clearance group and three of ten subjects in the RSV/A-delayed viral clearance group had a potential loss of an O-linked glycosylation motif. In comparison, one and six subjects gained O-linked glycosylation sites in the normal and delayed viral clearance groups, respectively. For RSV/B, two of five and five of eight individuals in the normal and delayed viral clearance groups, respectively, lost a potential O-linked glycosylation site. Whereas five and eight individuals in the normal and delayed clearance group, respectively, gained a potential O-linked glycosylation site. Notably, a variant T279I associated with a loss of a potential O-linked glycosylation site was found in at least seven RSV/B infected patients in both clearance groups. As expected, glycosylation motifs were not detected in the central conserved domain (CCD), which is a target for neutralizing antibodies. On the other hand, we detected variants in the heparin-binding domain (HBD), another conserved region of G protein which facilitates binding to heparin-sulfated proteoglycans on host cells (Feldman, Hendry, and Beeler 1999). We found one variant (I198T) with a gain of a potential O-linked glycosylation motif in the HBD that was present in three and six subjects in the normal and delayed clearance groups, respectively—Fig. 6A and Supplementary Table S1).

We next determined the changes in variants and in glycosylation sites of F protein at the initial visit. The F protein contains N-linked glycans, but it is not known to contain O-linked glycans even though it contains amino acids with the potential for O-linked glycosylation. The F protein contains several antigenic sites that can be recognized by neutralizing antibodies. Two of the major antigenic sites on the F protein are sites II and IV, which are well conserved. There were no non-synonymous variants found in these antigenic sites, although synonymous variants occurred (Fig. 6B and Supplementary Table S3). Missense variants were found in antigenic site V (L172Q) in six subjects infected with RSV/B and in site III (MP8a: L45F) in three subjects with RSV/B but did not cause any potential changes in N-linked glycosylation. All potential losses in N-linked glycosylations were observed in RSV/B isolates (Fig. 6B and Supplementary Table S3). A potential loss of N-glycosylation at N201S in antigenic site Øb was observed in three subjects (two in normal clearance and one the delayed clearance groups), and at N18H (outside any antigenic sites) in a single patient in the delayed clearance group (Fig. 5B and Supplementary Table S3). In RSV/A, a potential gain of N-glycosylation site at S105N was observed in one patient (Fig. 5B and Supplementary Table S3).

Among new intra-host variants, those appearing in patients after the first visit, we only detected one in the whole cohort causing a potential change affecting glycosylation sites in either G or F. It was observed in patient 813,024 (RSV/B delayed viral clearance), where a frameshift (visit 3—T216fs) occurred on a threonine residue causing a potential loss of an O-linked glycosylation site in the G protein (Supplementary Fig. S4 and Supplementary Table S1). For F protein, only two new missense variants were found, but they did not cause potential changes to glycosylation. They both appeared on visit 3 of patient 813,008 (RSV/A delayed viral clearance group) at positions T8S and C21Y and were not inside any known antigenic site (Supplementary Fig. S4 and Supplementary Table S4).

#### The effects of ribavirin treatments on the accumulation of variants in the L gene

**Figure 7.**
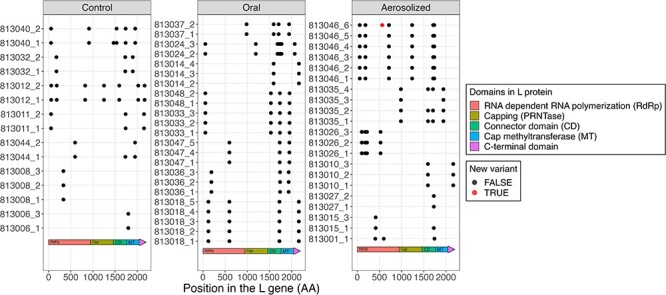
The effect of ribavirin treatment on the development of new variants on the polymerase (L) gene. Each panel represents a different treatment group with either no treatment (control) or treatment with oral or aerosol ribavirin. Only genomic differences that had a minimal 20 per cent allele frequency and 30× coverage were accepted as variants and are shown in this plot. All samples are represented as the patient number and visit number separated by an underscore on *y*-axis. Amino acid position on L gene and the protein domains are represented in the *x*-axis. The points represent non-synonymous variants, with red points representing variants that appeared after the first visit for that patient.

These RSV-infected HCT subjects were part of a randomized controlled trail of ribavirin, therefore, we wanted to investigate whether oral or aerosolized ribavirin treatment induced accumulation of variants in the L gene of RSV. Ribavirin is a nucleoside analog that inhibits RNA-dependent RNA polymerase (RdRp—gene L) and interferes with viral replication (Shook and Lin 2017). Ribavirin also affects the fidelity of the RNA polymerase, inducing the accumulation of new variants in RNA viruses upon treatment (Levi et al. 2010; Gnädig et al. 2012; Rozen-Gagnon et al. 2014). We observed many missense mutations in the L gene both in control and treated groups when compared to the reference genome, but all the missense mutations were present at enrollment and during subsequent visits, suggesting that the mutations were not induced by ribavirin (Fig. 7). Only one new variant was observed in the L gene in our entire cohort, a N564D missense variant, which can cause a potential loss of an N-linked glycosylation site. This variant emerged in the last visit of patient 813,046 (Fig. 7) approximately 28 days from enrollment and about 2 weeks after the end of the aerosol ribavirin treatment. Hence, our data suggest that neither aerosolized nor oral treatments induced resistance to ribavirin in the RSV-infected HCT adults.

## Discussion

In the present study we investigate the inter-host and intra-host variation of RSV/A and RSV/B in HCT adults who were part of a randomized clinical trial to evaluate oral versus aerosol ribavirin preemptive treatment. These adults either shed virus for >14 days (delayed viral clearance) and were associated with low humoral and mucosal antibody responses at 4 weeks post-enrollment or cleared the virus in <14 days (normal viral clearance) and mounted a robust antibody response at 4 weeks post-enrollment. To determine the genetic variability over time, we generated whole genome sequences of RSV isolates. We hypothesized that a ‘variant bottle neck effect’ would occur in the normal viral clearance group demonstrated by low overall variation because of their robust immune response and more rapid viral clearance. As a corollary, higher viral genomic variation would occur in the delayed viral clearance groups because of prolonged virus replication in an environment with reduced immune pressure. To our surprise, we observed similar average frequency of the various variant types in both normal and delayed viral clearance groups. However, there was the tendency for the delayed viral clearance groups to accumulate higher number of mutations. Two studies have described RSV variation in an immunocompromised host. Grad et al. were the first to report on the use of whole genome sequencing analysis to evaluate intra-host diversity of a single RSV/A isolate from a bone marrow transplanted infant with severe combined immune deficiency (Grad et al. 2014). Samples sequenced temporally over 75 days showed a significant increase in viral diversity which correlated with bone marrow engraftment. A second study in sixteen RSV-infected HCT adults with prolonged viral shedding, Sanger sequencing of the G gene showed identical sequences over the course of shedding period except for two subjects who developed a single amino acid substitution (Tabatabai et al. 2018). Our study is in line with the second study where the overall genetic diversity remained constant between normal and delayed viral clearance groups. Most of the RSV-infected HCT adults in the delayed viral clearance group stopped shedding virus within 30 days and this was most likely due to reconstitution of their immune system post-transplant.

We observed a trend towards greater intra-host variation in RSV/A and RSV/B-infected HCT adults in the delayed viral clearance groups as compared to normal viral clearance groups, although this was not statistical significance, likely due to the low number of subjects in our study. Both normal viral clearance groups were mostly stable in allelic frequencies in consecutive visits with some missense mutations occurring in the G gene. However, mixed variant populations developed after enrollment in the NS1, G, and L genes in 13 of 18 subjects in delayed viral clearance groups for RSV/A and B. It is possible that a low selective pressure created by the weakened immune system in the delayed viral clearance groups provides an environment for increased plasticity in RSV intra-host variation. Few studies have reported on the longitudinal variation of RSV during infection in healthy population. A study on fifty-three hospitalized infants demonstrated that the G gene was most variable and was the only gene with detectable positive selection (Do et al. 2015). Another study demonstrated that RSV/A exhibited greater within-host virus diversity than RSV/B in experimentally infected adults. Adults experimentally infected with RSV showed viral diversification at day 3, and mutations were concentrated in the M2 and NS2 genes (Lau et al. 2017). Contrary to experimentally infected adults, a study of 327 samples sequenced from a predominately infant population found that RSV/B exhibited greater within-host diversity than RSV/A, and variants were concentrated significantly in G gene. However, the intra-host variation in healthy infants was constant and did not show significant temporal changes (Lin et al. 2021). Like our results, intra-host variation in immunocompromised subjects showed greatest diversity in the G gene (Grad et al. 2014), (Tabatabai et al. 2018). The influence of viral copy number on accumulated variants showed minimal impact except for RSV/B-delayed, where we observed an increase in viral copy number (lower Ct value) that was associated with a rise in the number of variants. Although statistically significant, this correlation reflected subtle effects. Mild effects can still be meaningful and could potentially have implications, especially in the context of viral evolution or treatment responses. Further investigations could help in understanding the biological or clinical significance of this relationship.

Interestingly, in our cohort, two individuals only in the delayed clearance group accumulated variants with gain or loss of stop codons or displayed frame shifts in the G gene. Other studies have previously reported premature stop codon mutations in the G gene in RSV-infected immunocompromised individuals with prolonged viral shedding treated with intravenous immunoglobulin (IVIG) (Grad et al. 2014), (Tabatabai et al. 2018). In our study, only one individual was treated with IVIG (813,010); however, this individual did not demonstrate a premature stop codon in the G gene. All these studies, including ours, describe a set of common factors such as prolonged viral shedding and immune deficiency that were associated with truncated G proteins. It is unclear if the use of IVIG versus immune deficiency with prolonged viral shedding leads to selective pressure and results in truncations or frameshift mutations. In-vitro studies have reported that the use of monoclonal antibodies can generate premature stop codons, frameshift mutations, and missense mutations in the CCD of G protein and resistance to palivizumab (anti-F antibody) (García-Barreno et al. 1990; Rueda et al. 1991; Walsh, Falsey, and Sullender 1998; Adams et al. 2010). Not previously reported is the loss of the stop codon in the G gene detected in subject 813,026. Such a stop codon loss can result in the generation of a chimeric G-F protein. This variant persisted in three consecutive visits during the first 6 days post-enrollment suggesting that it maintained viral fitness. We also detected L172Q, and S173F/P missense mutations located in the prefusion antigenic site V of RSV/B F protein. These mutations have been described to cause resistance to Suptavumab, a monoclonal antibody which failed the phase III clinical trial due to development of community-emergent resistant mutations (Simões et al. 2021). L172Q in RSV F, was particularly widespread in our cohort, being found in 6/13 RSV/B isolates. In addition, Q209K mutation in the prefusion site Ø was found in four RSV/B isolates that can lead to resistance to site Ø monoclonal antibodies and have been recently described (Hammitt et al. 2022), (Lu et al. 2019).

A population comparison approach like the one we pursued here requires a common reference to be used. There are inherent risks to this approach, as not every position represents a common or rare allele of the entire RSV population. To overcome this problem, we utilized a consensus sequence across multiple RSV references. Furthermore, we required 30*×* coverage as a minimum together with 20 per cent allele frequency to avoid incorporating, e.g., sequencing errors as variants (false positives). However, it is important to keep in mind the drawbacks of reference-based approach with regards to variants or allele frequencies of variants. In our cohort, it appears that RSV/B/BA-infected subjects had a higher mean number of variants per individual compared to RSV/A/ON-infected subjects; these differences in mean variant numbers are likely due to the reference sequence used to compare the isolates. The reference for RSV/B/BA was assembled using a larger number of strains collected over 20 years. The RSV/A reference genome was assembled with fewer sequences collected from a 4-year period, hence the differences in variant numbers between RSV/A and RSV/B are likely due to an artifact of the reference used rather than a biological effect of viral evolution. Therefore, it is inadvisable to make direct comparisons of the inter-host variations between RSV/A and RSV/B data. Using an up-to-date reference would allow for direct comparison between RSV/A and RSV/B inter-host variant evolution. However, comparison of the intra-host variation between RSV/A and RSV/B can be performed.

Glycosylation dramatically influences the antigenicity of the G protein, contributing to immune evasion by masking G protein recognition by carbohydrate-specific antibodies. A potential new O-linked glycosylation site was described at position I279T in seven of the RSV/B-infected subjects distributed in both the normal and delayed viral clearance groups. Interestingly, all RSV/B had a potentially new O-linked glycosylation site (I198T) in the HBD of the G protein compared to the reference genome, possibly aiding in immune evasion. Glycosylation of the F protein was affected in only one RSV/A-infected patient, resulting in a potential gain of N-linked glycosylation site (S105N). In three RSV/B subjects, there was a potential loss of an N-linked glycosylation site at N201, within the prefusion antigenic site Ø. Changes in site Ø can affect the antigenic structure and binding of the new site Ø monoclonal antibody nirsevimab, which was recently approved and recommended in the United States for use in all infants less than 8 months of age entering their first RSV season (Hammitt et al. 2022). Lastly, treatment with oral or aerosol ribavirin did not induce any mutations in the L gene. This suggests that ribavirin does not cause accumulation of variants in the L gene and consequent drug resistance during short-term treatment.

In summary, we describe inter-host and intra-host variation of a unique population of HCT adults infected with RSV/A/ON or RSV/B/BA. The overall variation between the normal and delayed viral clearance groups remained constant. Variants that occurred were mostly identified in the G, F, and L genes. Intra-host variation was more pronounced in the delayed viral clearance groups, and unique structural variants impacting G protein structure were primarily detected only in adults with delayed viral clearance. Lastly, we consider that prolonged viral shedding and immune deficiency may drive some of these structural changes. It is therefore vital to sequence and monitor RSV isolates from immunocompromised subjects, as they can be a source of escape mutants entering the global community that can affect efficacy of first-ever RSV vaccines and the recently approved single-dose, long-acting monoclonal antibody, and have the potential to impact therapeutics in development.

## Methods

### Study design

We have previously reported on this open-label, block-randomized efficacy trial of ribavirin therapy of HCT adults with RSV infection at the time of enrollment limited to the upper respiratory tract (Ye et al. 2019), (Avadhanula et al. 2015). In brief, HCT recipients with laboratory-confirmed RSV URTI and negative chest radiography were enrolled within 72 hours of case ascertainment and stratified by level of risk (low to moderate or high) for progression to the lower respiratory tract. Eligible subjects were randomized to receive either 60 mg/ml of ribavirin 3 times per day for at least 5 days by aerosolization via a SPAG-2 generator or a onetime oral loading dose of 10 mg/kg then 20 mg/kg orally divided into three doses per day (max 1800 mg/day). The duration was for 10 days or longer at the discretion of the treating physician. A third group of RSV-infected HCT recipients were enrolled into an observational arm because they were at low risk of lower respiratory tract progression of their infection. None of the patients were given IVIG or palivizumab at the time of enrollment for treatment of their RSV URTI. If they progressed to develop LRTI, the primary treating physician at her/his discretion had the option to initiate therapy with aerosolized ribavirin with or without IVIG or palivizumab. Five individuals in our study faced treatment failure but none received IVIG. All five individuals were either in the RSV/A (813,025 and 813,047) or RSV/B (813,026, 813,030, and 813,035) delayed clearance groups. One other individual (813,010) in the RSV/B normal clearance group received IVIG. Demographics and clinical information were prospectively collected from structured interviews and review of medical records. Enrolled subjects were monitored for up to four weeks for signs and symptoms of upper and lower respiratory tract illness. The trial covered a 4-year period from January 2012 to April 2015. The institutional review board of the University of Texas MD Anderson Cancer Center and Baylor College of Medicine approved the study and informed consent was obtained from all the participants.

### Nasal wash sample collection and real-time reverse transcription polymerase chain reaction

Nasal wash samples were collected on all subjects at enrollment day 7 (± 1 day), day 14 (±1 day), and between day 21 and day 28 (±1 day) for detection of RSV by real-time, reverse-transcription polymerase chain reaction (RT-PCR). RSV/A and RSV/B were detected by two- step RT-PCR as described previously (Avadhanula et al. 2015).

### cDNA preparation for whole genome sequencing of RSV

RNA was analyzed using the RNA 6000 Nano assay (Agilent) or The RNA 6000 Pico assay for determination of an RNA Integrity Number (RIN) and DV200 metrics. cDNA was generated utilizing NEBNext® RNA First Strand Synthesis Module (E7525L; New England Biolabs Inc.) and NEBNext® Ultra™ II Directional RNA Second Strand Synthesis Module (E7550L; New England Biolabs Inc.). Total RNA in a 15 μl mixture containing random primers and 2X 1st strand cDNA synthesis buffer were incubated at 94°C for 10 min to fragment the RNA to 200–600bp. RNA was converted to cDNA by adding a 5 µl enzyme mix containing 500ng Actinomycin D (A7592, Thermo Fisher Scientific), 0.5 μl RNase inhibitor, and 1 μl of protoscript II reverse transcriptase, then incubated at 25°C for 10 min, 42°C for 50 min, and 70°C 15 min, before being cooled to 4°C on a thermocycler. Second strand cDNA was synthesized by adding a 60 μl of mix containing 48 μl H2O, 8 μl of 10X reaction buffer, and 4 μl of second strand synthesis enzyme, and incubated at 16°C for 1 hour on a thermocycler. The double strand (ds) cDNA were purified with 1.8X volume of AMPure XP beads (A63882, Beckman) and eluted into 42 μl 10 mM Tris buffer (Cat#A33566, Thermo Fisher Scientific). Because these libraries were prepared primarily for sequence capture, rRNA depletion, or Poly A+ RNA isolation steps were not performed.

### Library preparation *for whole genome sequencing of RSV*

The double-stranded cDNA was blunt-ended using NEBNext® End Repair Module (E6050L, NEB). Five microliter 10X End Repair (ER) reaction buffer and 5 μl ER enzyme were added to the ds cDNA. The ER reaction was incubated for 30 min at 20°C on a thermocycler. After the ER reaction, cDNA were purified with 1.8X volume AMPure XP beads and eluted into 42 μl nuclease free water (129,114, Qiagen). Next, 5 μl of 10X AT buffer and 3 μl of Klenow enzyme from NEBNext® dA-Tailing (AT) Module (E6053L, NEB) was added to the sample. The AT reaction was incubated at 37°C for 30 minutes. After incubation, samples were purified with 1.8X volume AMPure XP beads and eluted into 33 μl nuclease-free water (129,114, Qiagen). Illumina unique dual barcodes adapters (Cat# 20,022,370) were ligated onto samples by adding 2 μl of 5uM adapter, 10 μl 5X ligation buffer and 5 μl of Expresslink Ligase (A13726101, Thermo Fisher), and incubated at 20°C for 15 minutes. After adapter ligation, libraries were purified twice with 1.4X AMPure XP beads and eluted into 20 μl H2O. Libraries were amplified in 50 μl reactions containing 150 pmol of P1.1 (5ʹ-AATGATACGGCGACCACCGAGA) and P3 (5ʹ-CAAGCAGAAGACGGCATACGAGA) primer and Kapa HiFi HotStart Library Amplification kit (Cat# kk2612, Roche Sequencing and Life Science). The amplification was carried out at 95°C for 45 s, followed by 15 cycles of 95°C for 15 s, 60°C 30 s, and 72°C 1 minute, and 1 cycle at 72°C for 5 minutes. The amplified libraries were purified with 1.4X AMPure XP beads and eluted into 50 μl H2O. Quality assessment of the libraries was done using a Fragment Analyzer, DNA7500 kit (5067–1506, Agilent Technologies). The library yields were determined based on the 200–800-bp range.

#### Capture enrichment and sequencing

Pooled cDNA libraries were hybridized with biotin-labeled probes from the RSV Panel (Twist Biosciences, Inc) at 70°C for 16 hours as previously described (Doddapaneni et al. 2021). The RSV probe set size was 23.77 Mb and was designed based on 1,570 publicly available genomic sequences of RSV isolates. In this probe set, there are 87,025 unique probes of 80 bp length, which cover 99.79 per cent of the targeted isolates. Captured virus targets were incubated with streptavidin beads for 30 min at room temperature. Streptavidin beads bound with virus targets were washed and amplified with KAPA HiFi HotStart enzyme. The amount of each cDNA library pooled for hybridization and post-capture amplification PCR cycles (12 ∼ 20) were determined empirically according to the virus Ct values. In general, between 1.8–4.0 μg pre-capture library was used for hybridization with the probes and the post capture libraries were sequenced on Illumina NovaSeq S4 flow cell, to generate 2 × 150 bp paired-end reads.

#### Alignment and variant calling

After sequencing, adaptors were identified and removed and reads were trimmed for quality using Trimmomatic version 0.39–2 (Bolger, Lohse, and Usadel 2014) with the following parameters ‘ILLUMINACLIP:TruSeq3-PE.fa:2:30:10:2:keepBothReads LEADING:3 TRAILING:3 MINLEN:36’. Trimmed reads that were taxonomically classified using Kraken2 version 2.1.2 (Wood, Lu, and Langmead 2019) with default parameters. Reads were filtered using Kraken2 script extract_kraken_reads.py used with the parameters ‘-t 11244 -include-children’, with only reads classified as belonging to the Pneumoviridae family were kept. The filtered reads were then aligned to a reference genome. The reference genome for RSV/A/ON was generated using a consensus of an alignment of 81 RSV/A/ON sequences collected from April 2011 to July 2015. The reference for RSV/B/BA was generated using a consensus of an alignment of 266 RSV/B/BA sequences collected from April 1996 to January 2016. Sequence reads were first processed by a customized RSV module within Iterative Refinement Meta-Assembler v. 0.9.3 (https://github.com/XuetingQiu/ViralAssembly) (IRMA-RSV) (Shepard et al. 2016). IRMA-RSV was created by constructing the reference assembly templates for different subtypes and genotypes of RSV from publicly available nucleotide sequences. Briefly, both RSV/A/ON (ON) and RSV/B/BA (BA) whole-genome consensus sequences were retrieved from NCBI’s Nucleotide database (‘National Center for Biotechnology Information’ n.d.). Sequences with substantial missing regions or unresolved ‘N’s were excluded. These quality-filtered sequences were aligned with MUSCLE v. 3.8.31 (Edgar 2004) and built with the maximum likelihood phylogeny in RAxML v8.2 (Stamatakis 2014) (Stamatakis 2014) with an autoMRE option, for an efficient and automatic bootstrapping convergence criterion. The reference genomes available as FASTA files and the list of RSV isolates and their accessions numbers are listed in excel sheet in the supplemental materials. To independently assess the genotype of each sample independently, the ON and BA reference genomes were concatenated, and the resulting FASTA file was used as a reference genome for BWA MEM version 0.7.17-r1188 (Li and Durbin 2010) and then SAM files were converted to BAM, sorted, and indexed using samtools version 1.6 (Li et al. 2009). To detect variants on the BAM files, samtools mpileup (1.6) was used with the ‘-aa -A -d 0 -B -Q 0’ parameters, followed by iVar version 1.3.1 (Grubaugh et al. 2019) with standard parameters, using the same reference genome as the one used for alignment. The resulting TSV files generated by iVar were converted to VCF using the script ivar_variants_to_vcf.py from iVar. Lastly, variants were filtered for a minimal depth of 30 and minimal alternative allele frequency of 20 per cent using bcftools view (Danecek et al. 2021). Variant annotation was performed using snpEff version 4.3 u (Cingolani et al. 2012). Plots and subsequent analysis were generated using R and the ggplot package (Neuwirth 2014), (Yu et al. 2017).

### Statistical analysis

For the descriptive analysis, continuous variables were represented as a mean and standard deviation, and categorical variables were represented as frequencies or percentages. Frequencies were compared between groups using chi-square test or Fisher’s exact test. The difference in new intra-host variants that emerged after the first visit between the normal and delayed groups was tested using Fisher’s exact test. This test was applied for the number of new intra-host variants per group that occurred only after the first visit. Differences between mean Ct values between normal and delayed viral clearance groups by subtype were compared using unpaired two-tailed t test. To assess the relationship between the number of observed variants and viral load, measured in cycle threshold (Ct) values, Spearman’s correlation coefficient was employed. Differences between the mean number of variant types per group were assessed by using one-way ANOVA with Tukey’s post-test. Statistical significance was indicated for *p*-values <0.05. Statistical analyses were performed using R and in Excel.

## Supplementary Material

vead086_Supp

## Data Availability

The complete genomes of RSV from this study are being deposited in GenBank database with accession numbers OR466327-OR466401.
