# Supplementary material for: Inter and intra-host diversity of RSV in hematopoietic stem cell transplant adults with normal and delayed viral clearance
Source: Virus Evol. 2023 Dec 28;10(1):vead086. doi: 10.1093/ve/vead086 (PMC10868550; doi:10.1093/ve/vead086)
Supplement: vead086_Supp [file vead086_supp.zip › suppl_data/RSV A reference sequence file.docx]

>RSV/A/ON reference sequence

gtaaaccaaaaaaatggggcaaataagaatt

tggtaagtaccacttaaatttaactcctttggttagagatgggcagcaactcattgagta

tgataaaagttagattgcaaaatctgtttgacaatgatgaagtagcattgttaaaaataa

catgctatactgacaaattaatacagttaactaatgctttggctaaggcagttatacata

caatcaaattgaatggcattgtatttgtgcatgttattacaagtagtgatatttgcccta

ataataatattgtagtgaaatccaatttcacaacaatgccagtattacaaaatggaggtt

atatatgggaaatgatggaattaacacactgctctcaacctaatggcctaatagatgaca

attgtgaaattaaattctccaaaaaactaagtgattcaacaatgaccaattatatgaatc

aattatctgaattacttggatttgacctcaatccataaatcataataaatatcaactagc

aaatcaatgtcactaacaccattagttaatataaaacttgacagaagataaaaatggggc

aaataaatcaattcagccgacccaaccatggacacaacacacaatgataccacaccacaa

agactgatgatcacagacatgagaccattatcgcttgagactataataacatctctaacc

agagatatcataacacataaatttatatacttgataaatcatgaatgcatagtgagaaaa

cttgatgaaagacaggccacatttacatttctggtcaactatgaaatgaaactattgcac

aaagtgggaagcactaaatataaaaaatatactgaatacaacacaaaatatggcactttc

cctatgccaatatttatcaatcatgatgggttcttagaatgcattggcattaagcctacc

aagcacacacccataatatacaagtatgatctcaatccatgaatatcaaaccaagattca

aacaatccgaaataacaactttatgcataatcacactccatagtccaaatggagcctgaa

aattatagttatttaaaattaaggagagacataagatgaaagatggggcaaatacaaaaa

tggctcttagcaaagtcaagttgaatgatacactcaacaaagatcaacttctatcatcca

gcaaatataccatccaacggagcacaggagacagcattgacactcctaattatgatgtgc

agaaacacattaataagttatgtggcatgttattaatcacagaagatgctaatcataaat

tcactgggttaataggtatgttatatgctatgtctagattaggaagagaagacaccataa

aaatactcaaagatgcgggatatcatgttaaggcaaatggagtggatgtaacaacacatc

gtcaagacattaatgggaaagaaatgaaatttgaagtgttaacattagcaagcttaacaa

ctgaaattcaaatcaacattgagatagaatctagaaaatcctacaaaaaaatgctaaaag

aaatgggagaggtggctccagaatacaggcatgactctcctgattgtgggatgataatat

tatgtatagcagcattagtaataaccaaattagcagcaggagatagatcaggtcttacag

ctgtgattaggagagctaataatgtcctaaaaaatgaaatgaaacgttataaaggtttat

tacccaaggatatagccaacagcttctatgaagtgtttgaaaaatatcctcactttatag

atgtttttgttcattttggtatagcacaatcttctaccagaggtggcagtagagttgaag

ggatttttgcaggattgtttatgaatgcctatggtgcagggcaagtgatgttacggtggg

gggtcttagcaaaatcagttaaaaacattatgttaggacacgctagtgtacaagcagaaa

tggaacaagttgtggaggtgtatgagtatgctcagaaattgggtggagaagcaggattct

accatatattgaacaacccaaaagcatcactattatctttgactcaatttcctcacttct

ctagtgtagtattgggcaatgctgctggcctaggcataatgggagaatacagaggtacac

caaggaatcaagatttatatgatgctgcaaaagcatatgctgaacaactcaaagaaaatg

gtgtgattaactacagtgtattagatttgacagcagaagaactagaggctatcaaacatc

agcttaatccaaaagataatgatgtagagctttgagttaataaaaaggtggggcaaataa

atcatcatggaaaagtttgctcctgaattccatggagaagatgcaaacaacagagccacc

aaattcctagaatcaataaagggcaaattcacatcacccaaagatcccaagaaaaaagat

agtatcatatctgtcaactcaatagatatagaagtaaccaaagaaagccctataacatca

aattcaaccattataaacccaataaatgagacagatgatactgtagggaacaagcccaat

tatcaaagaaagcctctagtaagtttcaaagaagaccctacgccaagtgataatcctttt

tcaaaactatacaaagaaaccatagaaacatttgataacaatgaagaagaatctagctat

tcatatgaagaaataaatgatcagacaaacgataatataacagcaagattagataggatt

gatgagaaattaagtgaaatactaggaatgcttcacacattagtagtagcgagtgcagga

cccacatctgctcgggatggtataagagatgccatggttggtttaagagaagaaatgata

gaaaaaatcagaactgaagcattaatgaccaatgacagactagaagctatggcaagactc

aggaatgaagaaagtgaaaagatggcaaaagacacatcagatgaagtgtctctcaatcca

acatcagagaaactgaacaacctgttggaagggaatgatagtgacaatgatctatcactt

gaagatttctgattagctaccaaactgtacatcaaaacacaacaccaatagaaaaccaac

aaacaaaccaactcacccatccaaccaaacatctatctgctgattagccaaccagccaaa

aaacaaccagccaatctaaaactagccacccggaaaaaatcgatactatagttacaaaaa

aagatggggcaaatatggaaacatacgtgaataaacttcacgagggctccacatacacag

ctgctgttcaatacaatgtcctagaaaaagacgatgatcctgcatcacttacaatatggg

tgcccatgttccaatcatccataccagcagatctactcataaaagaactagccaatgtca

atatactagtgaaacaaatatccacacccaagggaccctcattaagagtcatgataaact

caagaagtgcagtgctagcacaaatgcccagcaaatttaccatatgtgccaatgtgtcct

tggatgaaagaagcaagctggcatatgatgtaaccacaccctgtgaaattaaggcatgca

gtctaacatgcctaaaatcaaaaaatatgttaactacagttaaagatctcactatgaaaa

cactcaacccaacacatgacatcattgctttatgtgaatttgaaaatatagtaacatcaa

aaaaagtcataataccaacatacctaagatctatcagcgtcagaaataaagatctgaaca

cacttgaaaatataacaaccactgaattcaaaaatgccattacaaatgcaaaaatcatcc

cttactcaggattactgttagtcatcacagtgactgacaacaaaggagcattcaaataca

taaagccacaaagtcaattcatagtagatcttggagcttacctagaaaaagaaagtatat

attatgttacaacaaattggaagcacacagctacacgatttgcaatcaaacccatggaag

attaacctttttcctctacatcaatgagtagattcatacaaactttctaactacattctt

cacttcacaatcataatcaccaaccctctgtggttcaatcaatcaaacaaaactcatcag

gagttccagatcatcccaagtcattgttcatcagatccagtactcaaataagttaataaa

aaatccacatggggcaaataatcattgagggaaatccaactaatcacaacatctgtcaac

atagacaagtcaacacgctagataaaatcaaccaatggaaaatacatccataactataga

attctcaagcaaattctggccttactttacactaatacacatgataacaacaataatctc

tttgataatcataatctccatcatgattgcaatactaaacaaactctgcgaatataatgt

attccataacaaaacctttgagctaccaagagctcgagtcaatacatagcattcaccaat

ctgatagctcaaaacagtaaccttgcatttgtaaatgaactaccctcacttcttcacaaa

accacatcaacatctcaccatgcaagccatcatctataccataaagtagttaattaaaaa

atagtcataacaatgaactaggatattaagaccaaaaacaacgctggggcaaatgcaaac

atgtccaaaaccaaggaccaacgcaccgccaagacactagaaaggacctgggacactctc

aatcatctattattcatatcatcgtgcttatacaagttaaatcttaaatctatagcacaa

atcacattatctattttggcaatgataatctcaacctcacttataattgcagccatcata

ttcatagcctcggcaaaccacaaagtcacactaacaactgcaatcatacaagatgcaacg

aaccagatcaagaacacaaccccaacatacctcacccagaatccccagcttggaatcagc

ttctccaatctgtccggaactacatcacaatccaccaccatactagcttcaacaacacca

agtgctgagtcaaccccacaatccacaacagtcaagatcaaaaacacaacaacaacccaa

atattacctagcaaacccaccacaaaacaacgccaaaataaaccacaaaacaaacccaac

aatgattttcactttgaagtgttcaattttgtaccctgcagcatatgcagcaacaatcca

acctgctgggccatctgcaagagaataccaaacaaaaaacctggaaagaaaaccaccacc

aagcccacaaaaaaaccaaccctcaagacaaccaaaaaagatcccaaacctcaaaccaca

aaaccaaaggaagtactcactaccaagcctacaggaaagccaaccatcaacaccactaaa

acaaacatcagaactacactgctcacctccaacaccaaaggaaatccagaacacacaagt

caagaggaaaccctccactcaaccacctccgaaggctatccaagcccatcacaagtctat

acaacatccggtcaagaggaaaccctccactcaaccacctccgaaggctatccaagccca

tcacaagtccatacaacatccgagtacctatcacaatctctatcttcatccaacacaaca

aaatgatagtcattaaaaagcgtattgttgcaaaaagccatgaccaaatcaaacagaatc

aaaatcaacactggggcaaataacaatggagttgccaatcctcaaaacaaatgctattac

cacaatccttgctgcagtcacactctgtttcgcttccagtcaaaacatcactgaagaatt

ttatcaatcaacatgcagtgcagttagcaaaggctatcttagtgctctaagaactggttg

gtatactagtgttataactatagaattaagtaatatcaaggaaaataagtgtaatggtac

agacgctaaggtaaaattaataaaacaagaattagataaatataaaaatgctgtaacaga

attgcagttgctcatgcaaagcacaccagcagccaacagtcgagccagaagagaactacc

aagatttatgaattatacactcaacaataccaaaaacaccaatgtaacattaagtaagaa

aaggaaaagaagatttcttggatttttgttaggtgttggatctgcaatcgccagtggcat

tgccgtatccaaggtcctgcacctagaaggggaagtgaacaaaatcaaaagtgctctact

atccacaaacaaggctgtagtcagcttatctaatggagtcagtgtcttaaccagcaaggt

gttagacctcaaaaactatatagataaacagttgttacctattgttaacaagcaaagctg

cagcatatcaaacattgaaactgtgatagagttccaacaaaagaacaacagactactaga

gattaccagagaatttagtgttaatgcaggtgtaactacacctgtaagcacttatatgtt

aactaatagtgagttattatcattaatcaatgatatgcctataacaaatgatcagaaaaa

gttaatgtccagcaatgttcaaatagttagacagcaaagttactctatcatgtcaataat

aaaagaggaagtcttagcatatgtagtacaattaccactatatggtgtaatagatactcc

ttgttggaaactacacacatcccctctatgtacaaccaacacaaaggaaggatccaacat

ctgcttaacaagaaccgacagaggatggtactgtgacaatgcaggatcagtatccttttt

cccacaagctgaaacatgtaaagttcaatcgaatcgggtgttttgtgacacaatgaacag

tttaacattaccaagtgaggtaaatctctgcaacattgacatattcaaccccaaatatga

ttgcaaaattatgacttcaaaaacagatgtaagcagctccgttatcacatctctaggagc

cattgtgtcatgctatggcaaaaccaaatgtacagcatccaataaaaatcgtgggatcat

aaagacattctctaacgggtgtgattatgtatcaaataagggggtggatactgtgtctgt

aggtaatacattatattatgtaaataagcaagaaggcaaaagtctctatgtaaaaggtga

accaataataaatttctatgatccattagtgttcccctctgatgaatttgatgcatcaat

atctcaagtcaatgagaaaattaatcagagtctagcatttatccgtaaatcagatgaatt

attacataatgtaaatgctggtaaatccaccacaaatatcatgataactaccataattat

agtaattatagtaatattgttagcattaattgcagttggactgcttctatactgcaaggc

cagaagcacaccagtcacattaagtaaggatcaactgagtggtataaataatattgcatt

tagtaactgaataaaaatagcacctaatcatattcttacaatggttcgctatttgaccat

agataacccatctatcattagattatcctaaaatttgaacttcatcacaactttcatcta

taaaccatctcacttacactttttaagtagattcctattttatagttatataaaacaatt

gaataccaaattaacttactatttgtaaaaatgagaactggggcaaatatgtcacgaagg

aatccttgcaaattcgaaattcgaggtcattgcttgaatggtaaaaggtgtcattttagt

cataattattttgaatggccaccccatgcactgcttgtaagacaaaactttatgttaaac

agaatacttaagtctatggataaaagcatagatactttgtcagaaataagtggagctgca

gagttggacagaacagaagagtatgccctcggtgtagttggagtgctagagagttatata

ggatcaataaataatataactaaacaatcagcatgtgttgccatgagcaaactccttact

gaactcaacagcgatgacatcaaaaaactaagggacaatgaagagccaaactcacccaaa

gtaagagtgtacaatactgtcatatcatatattgaaagcaacaggaagaacaataaacaa

actatccatctgttaaaaagattgccagcagacgtattgaagaaaaccatcaaaaacaca

ttggatatccacaagagcataaccatcaataacccaaaagaatcaactgttagtgatacg

aacgaccatgccaaaaataatgatactacctgacaaatatccttgtagtataaattccat

actaataacaagtaattgtagagtcactatgtataatcaaaaaaacacactatatatcaa

tcaaaacaaccaaaataaccatatatacccaccggatcaaccattcaatgaaatccattg

gacctctcaagacttgattgatgcaactcaaaattttctacaacatctaggtattactga

tgatatatacacaatatatatattagtgtcataatactcaatcctaatacttaccacatc

atcaaattattaactcaaacaattcaagctatgggacaaaatggatcccattattagtgg

aaattctgctaatgtttatctaactgatagttatttaaaaggtgttatttctttctcaga

atgtaacgctttaggaagttacatattcaatggtccttatctcaaaaatgattataccaa

cttaattagtagacaaaatccattaatagaacacataaatctaaagaaactaaatataac

acagtccttaatatctaagtatcataaaggtgaaataaaaatagaagaacctacttactt

tcagtcattacttatgacatacaagagtatgacctcgtcagaacagactactactactaa

tttacttaaaaagataataagaagagctatagaaatcagtgatgtcaaagtctatgctat

attgaataaactggggctcaaagaaaaagacaagattaaatccaataatggacaagatga

agacaactcagtcattactaccataatcaaagatgatatacttttagctgtcaaggataa

tcaatctcatcttaaagcagacaaaaatcaatccacaaaacaaaaagatacaatcaaaac

aacacttttgaagaaattaatgtgttcgatgcaacatcctccatcatggttaatacattg

gtttaatttatacacaaaattaaacagcatattaacacaatatcgatctagtgaggtaaa

aaaccatggttttatattgatagataatcatactcttagtggattccaatttattttgaa

tcaatatggttgtatagtttatcataaggaactcaaaagaattactgtgacaacttataa

tcaattcttgacatggaaagatattagccttagtagattaaatgtttgtttgattacatg

gattagtaactgtttgaacacattaaacaaaagcttaggcttaagatgtggattcaataa

tgttatcttgacacaattattcctttatggagattgtatactaaaactattccacaatga

ggggttctacataataaaagaggtagagggatttattatgtctctaattttgaatataac

agaagaagatcaattcagaaaacggttttataatagtatgctcaacaacatcacagatgc

cgccaacaaagctcaaaaaaatctgctatcaagagtatgtcatacattattagataagac

aatatcagataatataataaatggcagatggataattctattgagtaagttcctaaaatt

aattaagcttgcaggtgacaataacctcaacaatctgagtgaattatattttttgttcag

aatatttggacacccaatggtagatgaaagacaagccatggatgctgttaaagttaattg

caacgagaccaaattttacttgttaagtagtttgagtatgttaagaggagcttttatata

tagaattataaaagggtttgtaaataattacaacagatggcctactttaagaaatgccat

tgtcttacccttaagatggttaacttactataaactaaacacttatccttccttgttgga

acttacagaaagagatttgattgttctatcaggactacgtttctatcgagagtttcggtt

gcctaaaaaagtggatcttgaaatgatcataaatgataaggctatatcacctcctaaaaa

tttaatatggactagtttccctagaaattatatgccgtcacacatacaaaattatataga

acatgaaaaattaaaattctctgatagtgataaatcaagaagagtattagagtattattt

aagagataacaaattcaatgaatgtgatttacacaactgtgtagttaatcaaagttatct

taacaacccgaatcatgtggtatcattgacaggcaaagaaagagaactcagtgtaggtag

aatgtttgcaatgcaaccaggaatgttcagacaagttcaaatattagcagagaaaatgat

agcagaaaacatattacaatttttccctgaaagtcttacaagatatggtgatctagaact

acagaaaatattagaattgaaagcaggaataagtaacaaatcaaatcgttacaatgataa

ttacaacaattacattagtaagtgctctatcatcacagatctcagcaaattcaatcaagc

atttcgatatgaaacatcatgtatttgtagtgatgtactggatgaactgcatggtgtaca

atctctattttcctggttacatttaactattcctcatgtcacaataatatgcacatatag

gcatgcacccccctatataaaggatcatattgtagatcttaacaatgtagatgagcaaag

tggactatatagatatcatatgggtggtatcgaagggtggtgtcaaaaactatggaccat

agaagctatatcactattagatctaatatctctcaaagggaaattctcaattactgcttt

aattaatggtgacaatcaatcaatagatataagtaaaccagtcagactcatggaaggtca

aactcatgctcaagcagattatttgctagcattaaatagtctcaaattactgtataaaga

gtatgcaggaataggccacaaattaaaaggaactgagacttatatatcgagagatatgca

atttatgagtaaaacgatccaacataacggtgtatattacccagctagtataaagaaagt

cctaagagtgggaccgtggataaacactatacttgatgacttcaaagtgagtctagaatc

tataggtagtttgacacaagaattagaatatagaggtgaaagtctattatgcagtttaat

atttagaaatgtatggttatataatcaaattgcattacaacttaaaaatcatgcattatg

taacaacaaattatatttggatatattaaaagttctaaaacacttaaaaaccttttttaa

tcttgataacattgatacagcattaacattgtatatgaatttgcccatgttatttggtgg

tggtgatcccaacttgttatatcgaagtttctatagaagaactcctgatttcctcacaga

ggctatagttcactctgtgttcatacttagttattatacaaaccatgatttaaaagataa

acttcaagatctgtcagatgatagattgaataagttcttaacatgcataatcacgtttga

caaaaaccccaatgctgaattcgttacattgatgagagatcctcaagctttaggatctga

gaggcaagctaaaattactagcgaaatcaatagactggcagttaccgaggttttgagcac

agctccaaacaaaatattttccaaaagtgcacaacactataccactacagagatagatct

taatgatattatgcaaaatatagaacctacatatcctcacgggctaagagttgtttatga

aagtttacccttttataaagcagagaaaatagtaaatcttatatccggtacaaaatctat

aactaacatactggaaaagacttctgccatagacttaacagatattgatagagccactga

gatgatgaggaaaaacataactttgcttataaggatattaccattagattgtaacagaga

taaaagagaaatattgagtatggaaaacctaagtattactgaattaagcaaatacgttag

agaaagatcttggtctttatccaatatagttggtgttacatcacccagtatcatgtatac

aatggacataaaatatacaacaagcactatagctagtggcataatcatagagaaatataa

tgtcaacagtttaacacgtggtgagagaggacccactaaaccatgggttggttcatctac

acaagagaaaaagacaatgccagtttataatagacaagttttaaccaaaaaacagagaga

tcaaatagatctattagcaaaattggattgggtgtatgcatctatagataacaaggatga

atttatggaggaacttagcataggaactcttgggttaacatatgagaaggccaaaaaatt

attcccacaatatttaagtgttaactatttgcatcgtcttacagtcagtagtagaccatg

tgaattccctgcatctataccagcttatagaactacaaattatcactttgatactagccc

tattaatcgcatattaacagaaaagtatggtgatgaagatattgatatagtattccaaaa

ctgtataagctttggccttagcttaatgtctgtagtagaacaatttactaatgtatgtcc

taacagaattattctcatacccaagcttaatgagatacatttgatgaaacctcccatatt

cacaggtgatgttgatattcacaagttaaaacaagtgatacaaaaacaacatatgttttt

accagacaaaataagtttgactcaatatgtggaattattcttaagtaataaaacactcaa

atctggatctaatgttaattctaatttaatattggcgcataagatatctgactattttca

taatacttacattttaagtactaatttagctggacattggattcttattatacaacttat

gaaagattctaagggtatttttgaaaaagattggggagagggatatataactgatcatat

gttcattaatttgaaagttttcttcaatgcttataagacatatctcttgtgttttcataa

aggttacggcagagcaaagctggagtgtgatatgaatacttcagatctcctatgtgtatt

ggaattaatagacagtagttattggaagtctatgtctaaggtgtttttagaacaaaaagt

tatcaaatacattcttagccaggatgcaagtttacatagagtaaaaggatgtcatagctt

caaactatggtttcttaaacgtcttaatgtagcagaattcacggtttgcccttgggttgt

taacatagattatcatccaacacatatgaaagcaatattaacttatattgatcttgttag

aatgggattgataaatatagatagaatatacattaaaaataaacacaagttcaatgatga

gttttatacttctaatctgttttacattaattataacttctcagataatactcatctatt

aactaaacatataaggattgctaattccgaattagaaagtaattacaacaaattatatca

tcccacaccagaaaccctagaaaatatactaaccaatccggttaaaagtaatgaaaaaaa

gacactgagtgactattgtataggtaaaaatgttgactcaataatgttaccatcgttatc

taataagaagcttattaaatcgtctacaatgattagaaccaattacagcagacaagattt

gtataatttatttcctacggttgtgattgataaaattatagatcattcaggtaatacagc

caaatctaaccaactttacactactacttctcatcaaatatccttagtgcacaatagcac

atcactttattgcatgcttccttggcatcatattaatagattcaattttgtatttagttc

tacaggttgtaaaattagtatagagtatattttaaaagatcttaaaattaaggatcctaa

ttgtatagcattcataggtgaaggagcagggaatttattattgcgtacagtagtggaact

tcatcctgatataagatatatttacagaagtctgaaagattgcaatgatcatagtttacc

aattgagtttttaaggctgtacaatggacatatcaacattgattatggtgaaaatttgac

cattcctgctacagatgcaaccaacaacattcattggtcttatttacatataaagtttgc

tgaacctatcagtctttttgtctgtgatgctgaattgcctgtaacagtcaactggagtaa

gattataatagagtggagcaagcatgtaagaaaatgcaagtactgttcttcagttaataa

atgtacattaatagtaaaatatcatgctcaagatgatatcgatttcaaattagacaacat

aactatattaaaaacttatgtatgcttaggcagtaagttaaagggatctgaagtttactt

agtccttacaataggtcctgcaaatgtgttcccagtatttaatgtagtacaaaatgctaa

attgatactatcaagaaccaaaaatttcatcatgcctaaaaaagctgataaagagtctat

tgatgcaaatattaagagtttgataccctttctttgttaccctataacaaaaaaaggaat

taatactgcattgtctaaattaaagagtgttgttagtggagatatactatcatattctat

agctggacgtaatgaagttttcagcaataaacttataaatcataagcatatgaacatctt

aaagtggttcaatcatgttttaaatttcagatcaacagaattaaactataatcatttata

tatggtagaatctacttatcctcatctaagtgaattgttaaacagcttgacaaccaatga

acttaaaaaactgattaaaatcacaggtagtttgttatacaacttttataatgaataatg

agcaaaaatcttataacaaaaatagctacacactaacattgtattcaattatagttattt

aaaattaataattatataatttttaataacttctagtgaactaatcctaaaattatcatt

ttgatctaggaagaataagtttaaatccaaatctaattggtttatatgtatattaactaa

attacgagatattagtttttg
