# Supplementary material for: Inter and intra-host diversity of RSV in hematopoietic stem cell transplant adults with normal and delayed viral clearance
Source: Virus Evol. 2023 Dec 28;10(1):vead086. doi: 10.1093/ve/vead086 (PMC10868550; doi:10.1093/ve/vead086)
Supplement: vead086_Supp [file vead086_supp.zip › suppl_data/RSV B reference sequence file.docx]

RSV/B/BA reference sequence

acgcgaaaaaatgcgtactacaaacttgcacactcgaaaaaaatggggcaaataagaatt

tgataagtgctatttaagtctaacctttttaatcagaaatggggtgcaattcactgagca

tgataaaggttagattacaaaatttatttgacaatgacgaagtagcattgttaaaaataa

catgttatactgacaaattaattcttctgactaatgcattagccaaagcaacaatacata

caattaaattaaacggcatagtttttatacatgttataacaagcagtgaagtgtgccctg

ataacaatattgtagtgaaatctaactttacaacaatgccaatattacaaaatggaggat

acatatgggaattgattgaattgacacactgctctcaattaaatggtctaatagatgata

attgtgaaatcaaattttctaaaagactaagtgactcagtaatgactgattatatgaatc

aaatatctgatttacttgggcttgatctccattcatgaattatgtttagtctaattcaat

agacatgtgtttattaccattttagttaatataaaacctcatcaaagggaaatggggcaa

ataaactcacccaatcaatcaaaccatgagcactacaaacgacaacaccaccatgcaaag

attgatgatcacagacatgagacccctgtcgatggattcaataataacatctctcaccaa

agaaatcatcacacacaaattcatatacttgataaacaatgaatgtattgtaagaaaact

cgatgaaagacaagctacatttacattcctagtcaattatgagatgaagctattgcacaa

agtagggagtaccaaatacaagaaatacactgaatataatacaaaatatggcacattccc

tatgcctatatttatcaatcatggcgggtttctagaatgtattggcattaagcctacaaa

acatactcctataatatacaaatatgacctcaacccgtaacttccaacaaaaaaaccaac

tcatccaaaccaagctattctctaaacaacagtgctcaacagttaagaaggagctaatcc

attttagtaattaaaaataagggtgaagccagtaacataaattggggcaaatacaaagat

ggctcttagcaaagtcaagttgaatgatacattaaataaggatcagctgctgtcatccag

caaatacactattcaacgtagtacaggagataatattgacactcccaattatgatgtgca

aaaacacctaaacaaactatgtggtatgctattaatcactgaagatgcaaatcataaatt

cacaggattaataggtatgctatatgctatgtccaggttaggaagggaagacactataaa

gatacttaaagatgctggatatcatgttaaagctaatggagtagatataacaacatatcg

tcaagatataaatggaaaggaaatgaaattcgaagtattaacattatcaagcttgacatc

agaaatacaagtcaatattgagatagaatctagaaagtcctacaaaaaaatgctaaaaga

gatgggagaagtggctccagaatataggcatgattctccagactgtgggatgataatact

gtgtatagctgcccttgtaataaccaaattagcagcaggagatagatcaggtcttacagc

agtaattaggagggcaaacaatgtcttaaaaaacgaaataaaacgctacaagggcctaat

accaaaggacatagccaacagtttttatgaagtgtttgaaaaacaccctcatcttataga

tgtttttgtgcactttggcattgcacaatcatccacaagagggggtagtagagttgaagg

aatctttgcaggattgtttatgaatgcctatggttcaggacaagtaatgctaagatgggg

agttttagccaaatctgtaaaaaatatcatgctaggacatgctagtgtccaagcagaaat

ggagcaagttgtggaagtctatgagtatgcacagaagttgggaggagaagctggtttcta

ccatatattgaacaatccaaaagcatcattgctgtcattaactcaatttcctaacttctc

aagtgtggtcctaggcaatgcagcaggtctaggcataatgggagagtatagaggtacacc

aagaaaccaagatctctatgatgcagccaaagcatatgcagagcaactcaaagaaaatgg

agtaataaactacagtgtattagacttaacaacagaagaattggaagccataaagcatca

actcaaccccaaagaagatgacgtagagctttaagttaacaaaaaatacggggcaaataa

gtcaacatggagaagtttgcacctgaatttcatggagaagatgcaaataacaaagctacc

aaattcctagaatcaataaaaggcaagtttgcatcatccaaagatcctaagaagaaagat

agcataatatctgtcaactcaatagacatagaagtcactaaagagagcccgataacatct

ggcaccaacattatcaatccaacaagtgaagccgacagtaccccagaaaccaaagccaac

tacccaagaaaacccctagtaagcttcaaagaagatctcaccccaagtgataaccctttc

tctaagttgtacaaagaaaccatagaaacatttgataacaatgaagaagaatctagctac

tcatatgaggagatcaatgatcaaacaaatgacaacattacagcaagactagatagaatt

gatgaaaaattaagtgaaatattaggaatgctccatacattagtagttgcaagtgcagga

cctacttcggctcgtgatggaataagagatgctatggttggtctaagagaagaaatgata

gaaaaaataagagcagaagcattaatgaccaatgataggttagaggctatggcaagactt

aggaatgaggaaagcgaaaaaatggcaaaagacacctcagatgaagtgtctctcaatcca

acctccaaaaaattgagtgacttgttggaagacaacgatagcgacaatgatctatcactt

gatgatttttgatcagtgatcaactcactcagcaatcaacaacatcaataaaacagacac

caatccattgaatcaattgccagactgaaaaaacaaacatccatcagcagaaccaccaac

caatcaatcaaccaattgatcaatcagcaccctgacaaaattaacaatatagtaacaaaa

aaagaacaagatggggcaaatatggaaacatacgtgaacaagcttcacgaaggctccaca

tacacagcagctgttcagtacaatgttctagaaaaagatgatgatcccgcatcactaaca

atatgggtgcctatgttccagtcatctgtgccagcagacttgctcataaaagaacttgca

agcatcaacatactagtaaagcagatctctacgcccaaaggaccttcactacgagtcacg

atcaactcaagaagtgctgtgctggctcaaatgcctagtaatttcaccataagtgcaaat

gtatcattagatgaaagaagcaaattagcatatgatgtaactacaccttgtgaaatcaaa

gcatgcagtctaacatgcttaaaagtaaaaagtatgttaactacagtcaaagatctaacc

atgaagacattcaaccccactcatgagatcattgctctatgtgaatttgaaaatattatg

acatcaaaaagagtaataataccaacctatctaagatcaattagtgtcaaaaacaaggac

ctgaactcactagaaaatatagcaaccaccgaattcaaaaatgctatcaccaatgctaaa

attattccctatgcaggattagtattagttatcacggttactgataataaaggagcattc

aaatatatcaagccacagagtcaatttatagtggatcttggtgcctacctagaaaaagag

agcatatattatgtgactactaattggaagcatacagctacacgtttttcaatcaaacca

ctagaggattaaacccaattatcaacattgaatgacaggttcacatatatcctcaactgc

acactatatctaaacatcataaacatctacactacacacttcatcacacaaaccaatccc

actcaaaatccaaaatcacttccagccattgtctgccagacctagagtgcgaataggtaa

ataaaaccagaatatggggtaaatagatatcagttagagttcaatcaatctcaacaacca

tctataccgccaattcaatacatatactgcaaatctcaaaatgggaaacacatccatcac

aatagaattcacaagcaaattttggccctattttacactaatacatatgatcttaactct

aatctctttactaattataatcactattatgattgcaatactaaataagctaagtgaaca

taaaacattctgtaacaaaactcttgaacaaggacagatgtatcaaatcaacacatagtg

ttctcccattatgctgtgtcaaattataatcttgtatatataaataaacaaatccaatct

tctcacagagtcatggtatcacaaaaccatgccaaccatcatggtagcatagagtagtta

tttaaaaattaacataatgatgaattattagtatgggatcaaaaacaacattggggcaaa

tgcaaccatgtccaaaaacaagaatcaacgcactgccaggactctagaaaagacctggga

tactcttaatcatctaattgtaatatcctcttgtttatacaaattaaatttaaaatctat

agcacaaatagcactatcagttttggcaatgataatctcaacctctctcataattgcagc

cataatattcatcatctctgccaatcacaaagttacactaacaactgtcacagttcaaac

aataaaaaaccacactgagaaaaacataaccacttaccttactcaagtctcaccagaaag

ggttagcccatccaaacaacccacaaccacaccaccaatccacacaaactcagccacaat

atcacctaatacaaaatcagaaacacaccatacaacagcacaaaccaaaggcagaacctc

tactccaacacagaacaacaagccaagcacaaaaccacgtccaaaaaatccaccaaaaaa

agatgattaccattttgaagtgttcaacttcgttccctgtagtatatgtggcaacaatca

actctgcaaatccatttgcaaaacaataccaagcaataaaccaaagaaaaaaccaaccat

aaaacccacaaacaaaccacccaccaaaaccacaaacaaaagagacccaaaaacactagc

caaaacaccgaaaaaagaaaccaccattaacccaacaaaaaaaccaacccccaagaccac

agaaagagacaccagcaccccacaatccactgtgctcgacacaaccacatcaaaacacac

agaaagagacaccagcacctcacaatccattgcgcttgacacaaccacatcaaaacacac

aatccaacagcaatccctctactcaaccacccccgaaaacacacccaactccacacaaac

acccacagcatccgagccctccacatcaaattccacctaaaaactccagtcatatgctta

gttatttaaaaactacatcttagcagagaaccgtgatccctcaagcaagaacgaaattaa

atctggggcaaataaccatggagttgctgatccatagatcaagtgcaatcttcctaactc

ttgctattaatgcattgtacctcacctcaagtcagaacataactgaggagttttaccaat

cgacatgtagtgcagttagcagaggttacttgagtgctttaagaacaggttggtatacca

gtgtcataacaatagaattaagtaatataaaagaaaccaaatgcaatggaactgacacta

aagtaaaacttataaaacaagaattagataagtataagaatgcagtaacagaattacagt

tacttatgcaaaacacaccagctgccaacaaccgggccagaagagaagcaccacagtata

tgaactacacaatcaataccactaaaaacctaaatgtatcaataagcaagaagaggaaac

gaagatttctgggcttcttgttaggtgtaggatctgcaatagcaagtggtatagctgtat

ccaaagttctacaccttgaaggagaagtgaacaagatcaaaaatgctttgctgtctacaa

acaaagctgtagtcagtctatcaaatggggtcagtgttttaaccagcaaagtgttagatc

tcaagaattatataaacaaccaattattacctatagtaaatcaacagagttgtcgcattt

ccaacattgaaacagttatagaattccagcagaagaacagcagattgttggaaatcacca

gagaatttagtgtcaatgcaggtgtaacgacacctttaagcacttacatgttaacaaaca

gtgagttactatcattaatcaatgatatgcctataacaaatgatcagaaaaaattaatgt

caagcaatgttcagatagtaaggcaacaaagttattctatcatgtctataataaaggaag

aagtccttgcatatgttgtacagctacctatctatggtgtaattgatacaccttgctgga

aattacacacatcacctctgtgcaccaccaacatcaaagaaggatcaaatatttgtttaa

caaggactgatagaggatggtactgtgataatgcaggatcagtatccttctttccacagg

ctgacacttgtaaagtacagtccaatcgagtattttgtgacactatgaacagtttgacat

taccaagtgaagtcagcctttgtaacactgacatattcaattccaagtatgactgcaaaa

ttatgacatcaaaaacagacataagcagctcagtaattacttctctaggagctatagtgt

catgctatggtaaaactaaatgcactgcatccaacaaaaatcgtggaattataaagacat

tttctaatggttgtgattatgtgtcaaacaaaggagtagatactgtatcagtgggcaaca

ctttatactatgtcaacaagctggaaggcaaaaacctttatgtaaaaggggaacctataa

taaattactatgaccctctagtgtttccttctgatgagtttgatgcatcaatatctcaag

tcaatgaaaaaattaatcaaagtttagcttttattcgtagatccgatgaattattacata

atgtaaatactggaaaatctactacaaatattatgataactgcaattattatagtaatca

ttgtagtattgttatcattaatagctattggtttactgttgtattgcaaagccaaaaaca

caccagttacactaagcaaagaccaactaagtggaatcaataatattgcattcagcaaat

agacaaaaaaccacctgatcatgtttcaacaacaatctgctgaccaccaatcccaaatca

acttacaacaaatacttcaacatcacagcacaggctgaatcatttcctcgcatcatgcta

cccacacaactaagctagatccttaactcatagttacataaaaacctcaaatatcgcaat

caacactaaatcaacacatcattcacaaaactaacagctggggcaaatatgtcgcgaaga

aatccctgcaaatttgagattagaggtcattgcttgaatggtagaagatgccactacagt

cataattactttgaatggcctcctcatgcattactagtgaggcaaaacttcatgttaaac

aagatactcaagtcaatggacaagagcatagacactttgtctgaaataagtggagctgct

gaacttgatagaacagaagaatatgctcttggtatagttggagtgctagagagttacata

ggatctataaacaacataacaaaacaatcagcatgtgttgctatgagtaaacttcttatt

gagatcaatagtgatgacattaaaaagctgagagacaatgaagaacccaattcacctaag

ataagagtgtacaatactgttatatcatacatcgagagcaatagaaaaaacagcaagcaa

accatccatctgctcaaacgattaccagcagacgtgctgaagaagacaataaagaacaca

ttagatatccacaaaagcataaccataagcaacccaaaagagtcaaccgtaagtgatcaa

aatgaccaaaccaaaaataatgatattaccggataaatatccttgtagtatatcatccat

attgatctcaagtgaaagcatgattgctacattcaatcataaagacatattacaatttaa

ccacaaccatttggataaccaccagtgtttattaaatcatatatttgatgaaattcattg

gacacctaaaaacttattagatgccactcaacaatttctccaacatcttaacatccctga

agatatatatacagtatatatattagtgtcataatgcttgatcataacgattctatatca

tccaaccataaaactgtcttaataaggttatgggacaaaatggatcccattattaatgga

agctctgctaatgtatatctaactgatagttatctaaaaggtgttatctctttttcagaa

tgtaatgctttagggagttacctttttaacggcccttatcttaaaaatgattataccaac

ttaattagtagacaaagcccactactagagcatatgaatctaaaaaaactaactataaca

cagtcattaatatctagataccataaaggtgaactgaaattagaagaaccaacttatttc

cagtcattacttatgacatataaaagcatgtcctcgtctgaacaaattgctacaactaac

ttacttaaaaaaataatacgaagagctatagaaataagtgatgtaaaggtgtacgccatc

ttgaataaactaggactaaaggaaaaggacagagttaagcccaacaacaattcaggtgat

gaaaactcagtacttacaaccataattaaagatgatatactctcagctgtggaaaacaat

caatcatatacaaattcagacaaaaattactcagtaaatcaaaatatcaatatcaaaaca

acactcttaaaaaaattgatgtgttcaatgcaacatcctccatcatggttaatacactgg

ttcaatttatatacaaaattaaataacatattaacacaatatcgatcaaatgaggtaaaa

agtcatgggtttatattaatagataatcaaactttgagtggttttcagtttattttaaat

caatatggttgcattgtttatcataaagggctcaaaaaaatcacaactactacatacaat

caatttttgacatggaaagacatcagccttagcagattaaatgtttgcttaattacttgg

ataagtaattgtttaaatacattaaataaaagcttagggttgagatgcggattcaataat

gttgtgctatcacaattattcctttatggagattgtatactgaaattatttcataatgaa

ggcttttacataataaaagaagtagaaggatttattatgtctttaattctaaacataaca

gaagaagatcaatttaggaaacgattttataatagcatgctaaataacatcacagatgca

gctattaaggctcaaaaggacctactatcaagagtatgtcacactttattagacaagaca

gtgtctgataatatcataaatggtaaatggataattctattaagtaaatttcttaaattg

attaagcttgcaggtgataataatctcaataacttgagtgagctatattttctcttcaga

atctttggacatccaatggttgatgaaagacaagcaatggatgctgtaagaattaactgc

aatgaaactaagttctatttattaagtagcctaagtacgttgagaggtgctttcatttat

agaatcataaaagggtttgtaaatacctacaacagatggcccactttaaggaatgctatt

gtcctacctctaagatggttaaactattataaacttaatacttatccatctctacttgaa

atcacagaaaatgatttgattattttatcaggattgcggttctatcgtgagtttcatctg

cctaaaaaagtggatcttgaaatgataataaatgacaaagctatttctcctccaaaagat

ctaatatggactagttttcctagaaattacatgccatcacatatacaaaattatatagaa

catgaaaagttgaagttctctgaaagcgacagatcaagaagagtactagagtattacttg

agagataataaattcaatgaatgtgatctatacaattgtgtagttaatcaaagctatctc

aacaactctaatcatgtggtatcactaactggtaaagaaagagagctcagtgtgggtaga

atgtttgctatgcaaccaggtatgtttaggcaaatccaaatcttagcagagaaaatgata

gccgaaaatattttacaattcttccctgagagtttgacaagatatggtgatctagagctt

caaaagatattagaattaaaagcaggaataagcaacaagtcaaatcgttataatgataac

tacaacaattatatcagtaaatgttctataataacagatcttagcaaatttaatcaagca

tttagatatgaaacatcatgtatctgcagtgatgtattagatgaactgcatggggtacaa

tctctattctcttggttgcatttaacaatacctcttgtcacaataatatgtacatataga

catgcacctccttttataaaggatcatgttgtcaatcttaatgaagttgatgaacaaagt

gggttatacagatatcatatgggtggtattgagggctggtgtcaaaaactgtggaccatt

gaagccatatcattattagatctaatatctcttaaaggtaaattctccatcacagctctg

ataaatggtgataatcagtcaattgatataagtaaaccagttagacttatagagggtcag

acccatgctcaagcagattatttgttagcattaaatagccttaaattgctatataaagag

tatgcaggcataggccataagcttaagggaactgagacctatatatcccgagatatgcag

ttcatgagcaaaacaatccagcacaatggagtgtactatccagccagtatcaaaaaagtc

ctgagagtaggtccatggataaatacaatacttgatgattttaaagttagtttagaatct

ataggtagcttaacacaggagttagaatacagaggagaaagcttattatgcagtttaata

tttaggaacatttggttatacaatcaaattgctctgcaactccgaaatcatgcattatgt

aataataagctatatttagatatattgaaagtattaaaacacttaaaaaccttttttaat

cttgatagtatcgatacggcgttatcattgtatatgaacttgcctatgctgtttggtggt

ggtgatcctaatttgttatatcgaagcttttataggagaactccagacttccttacagaa

gctatagtacattcagtgttcgtgttgagctattatactggtcacgatctacaagataag

ctccaggatcttccagatgatagactgaacaaattcttgacttgtgtcatcacatttgat

aaaaatccaaatgccgagtttgtaacattgatgagggatccacaggctttagggtctgaa

aggcaagctaaaattactagtgagattaatagattagcagtaacagaagtcttaagtata

gctccaaacaaaatattttctaaaagtgcgcaacactatactaccactgagattgatcta

aatgacattatgcaaaatatagaaccaacttaccctcatggattaagagttgtttatgaa

agtttacctttttataaagcagaaaaaatagttaatcttatatcaggaacaaaatccata

actaatatacttgaaaaaacatcagcgatagatacaactgatattaatagggctactgat

atgatgaggaaaaatataaccttacttataaggatacttccactagattgtaacaaagac

aaaagagagttattaagtttagaaaatcttagcataactgaattaagcaagtatgtaaga

gaaagatcttggtcattatccaatatagtaggagtaacatcgccaagtattatgttcaca

atggacattaaatatacaactagcactatagccagtggtataattatagaaaaatataat

gttaatggtttaactcgtggtgaaagaggacctactaagccatgggtaggttcatctacg

caggagaaaaaaacaatgccagtgtacaatagacaagttttaaccaaaaagcaaagagac

caaatagatttattagcaaaattagactgggtatatgcatccatagacaacaaagatgaa

ttcatggaagaactgagtactggaacacttggactgtcatatgaaaaagccaaaaagttg

tttccacaatatctaagtgtcaattatttacaccggttaacagtcagtagtagaccatgc

gaattccctgcctcaataccagcttatagaacaacaaattatcattttgatactagtcct

atcaatcatgtattaacagaaaagtatggagatgaagatatcgacattgtgtttcaaaat

tgcataagttttggtcttagcttgatgtcagttgtggaacaattcacaaacatatgtcct

aatagaattattctcataccgaagctgaatgagatacatttgatgaaacctcctatattt

acaggagatgttgatatcatcaagttgaagcaagtgatacaaaaacagcatatgttccta

ccagataaaataagtttaacccaatatgtagaattattcttaagtaacaaagcacttaaa

tctggatcccacatcaactctaatttaatattagtacataaaatgtctgattattttcat

aatgcgtatattttaagtactaatttagctggacattggattctgattattcaacttatg

aaagattcaaaaggtatttttgaaaaagattggggagaggggtatataactgatcatatg

ttcattaatttgaatgttttctttaatgcttataagacttatttgctatgttttcataga

ggttatggtaaagcaaaattagaatgtgatatgaacacttcagatcttctttgtgttttg

gagttaatagacagtagctactggaaatctatgtctaaagttttcctagaacaaaaagtc

ataaaatacatagtcaatcaagacacaagtttgcatagaataaaaggctgtcacagtttt

aagttgtggtttttaaaacgccttaataatgctaaatttaccgtatgcccttgggttgtt

aacatagattatcacccaacacacatgaaagctatattatcttacatagatttagttaga

atggggttaataaatgtagataaattaaccattaaaaataaaaacaaattcaatgatgaa

ttttacacatcaaatctcttttacattagttataacttttcagacaacactcatctgcta

acaaaacaaataagaattgctaattcagaattagaagataattataacaaactatatcac

ccaaccccagaaactttagaaaatatatcattaatccctgttaaaagtaataatagaaac

aaacctaaattttgtataagtggaaatactgaatctatgatgacgtcaacattctctaat

aaaatgcatattaaatcttccactgttaccacaagattcaattatagcagacaagacttg

tacaatttatttccaattgttgtgatagacaggattatagatcattcaggtaatacagaa

aaatctaaccaactttacaccaccacttcacatcagacatctttagtaaggaatagtgca

tcactttattgcatgcttccttggcatcatgtcaatagatttaactttgtatttagttcc

acaggatgcaagatcagtatagagtatattttaaaagatcttaagattaaagatcccagt

tgtatagcattcataggtgaaggagctggtaacttattattacgtacggtagtagaactt

catcctgacataagatatatttacagaagtttaaaagattgcaatgatcatagtttacct

attgaatttctaaggttatacaacgggcatataaacatagattatggtgagaatttaacc

attcctgctacagatgcaactaacaacatacattggtcttatttacatataaaatttgca

gaacctattagtatctttgtctgcgatgctgaattacctgtcacagccaattggagtaaa

attataattgaatggagtaagcatgtaagaaagtgcaaatactgttcttctgtaaataga

tgcattttaattgcaaaatatcatgctcaagatgatattgatttcaaattagataacatt

actatattaaaaacttatgtgtgcctaggtagcaagttaaaaggatctgaagtttactta

gtccttacaataggccctgcaaatatacttcctgtttttgatgttgtgcaaaatgctaaa

ttgattctttcaagaactaaaaatttcattatgcctaaaaagattgacaaggaatctatc

gatgcaaatattaaaagcttaatacctttcctttgttaccctataacaaaaaatggaatt

aagacttcattgtcaaaattgaagagtgtagttaatggagatatattatcatattctata

gctggacgtaatgaagtattcagcaacaagcttataaaccacaagcatatgaatatctta

aaatggctggatcatgttttaaactttagatcagctgaacttaattacaatcatttatac

atgatagagtccacatatccttacttgagtgaattgttaaatagtttaacaaccaatgag

ctcaagaagctgattaaaataacaggtagtgtactatacaaccttcctaatgaacagtaa

cttaaaatatcattaacaagtttggtcaaatttagatgctaacacatcattatattatag

ttattaaaaaatatgcaaacttttcaataatttagcatattgattccaaaattatcattt

tagtcttaagggattaaataaaagtctaaaactaacaattacacatgtgcatttacaaca

caacgagacattagtttttgacacttttttctcgttattaacaagtactccaggtcattc

tga
