# Supplementary material for: Inter and intra-host diversity of RSV in hematopoietic stem cell transplant adults with normal and delayed viral clearance
Source: Virus Evol. 2023 Dec 28;10(1):vead086. doi: 10.1093/ve/vead086 (PMC10868550; doi:10.1093/ve/vead086)
Supplement: vead086_Supp [file vead086_supp.zip › suppl_data/Suppl Tables 2-4.docx]

**Supplemental Table** ***1A)*** A list containing all variants found. If a variant is found in multiple isolates, each row represents a combination of variant and visit of each subject containing specific information for that combination. **1B)** A simplified version of suppl. Table 1A, containing information about population distribution of each variant, where each variant appears in a single row*.*

*# SEE EXCEL FILE*

**Supplemental Table 2)** A list of the most widespread variants in our cohort by number of subjects.

| **Reference genome** | **Position** | **Gene** | **Type of mutation** | **Variant** | **Reference genome base** | **Variant base** | **Type of mutation** | **Potential change in glycosylation site** | **Number of samples with this variant** | **Number of unique**  **subjects with this variant** |
| --- | --- | --- | --- | --- | --- | --- | --- | --- | --- | --- |
| RSV/B/BA | 5280 | G | SNV | I198T | T | C | missense | O-linked site gained | 26 | 9 |
| RSV/B/BA | 5523 | G | SNV | I279T | T | C | missense | O-linked site gained | 21 | 7 |
| RSV/B/BA | 1784 | N | SNV | H216Y | C | T | missense | No changes | 17 | 6 |
| RSV/B/BA | 4446 | SH | SNV | T49I | C | T | missense | O-linked site lost | 18 | 6 |
| RSV/B/BA | 6025 | F | SNV | A103V | C | T | missense | No changes | 18 | 6 |
| RSV/B/BA | 6232 | F | SNV | L172Q | T | A | missense | No changes | 18 | 6 |
| RSV/B/BA | 8209 | M2-1 | SNV | V181I | G | A | missense | No changes | 17 | 6 |
| RSV/B/BA | 8725 | L | SNV | L56I | C | A | missense | No changes | 17 | 6 |
| RSV/B/BA | 13766 | L | SNV | N1736S | A | G | missense | N-linked site lost | 17 | 6 |
| RSV/A/ON | 3383 | M | SNV | I43M | A | G | missense | No changes | 28 | 11 |
| RSV/A/ON | 5501 | G | SNV | P274L | C | T | missense | No changes | 22 | 9 |
| RSV/A/ON | 5573 | G | SNV | P298L | C | T | missense | No changes | 20 | 8 |
| RSV/A/ON | 5590 | G | SNV | H304Y | C | T | missense | No changes | 20 | 8 |

**Supplemental Table 3)** A list of all variants in the F gene potentially affecting glycosylation sites.

| **Reference genome** | **Position** | **Amino acid substitution** | **Reference base** | **Variant**  **base** | **Potential change in glycosylation** | **Number of samples with this variant** | **Number of subjects with this variant** | **F antigenic site** |
| --- | --- | --- | --- | --- | --- | --- | --- | --- |
| RSV/B/BA | 5769 | N18H | A | C | N-linked site lost | 2 | 1 | No |
| RSV/B/BA | 5772 | A19T | G | A | O-linked site gained | 2 | 1 | No |
| RSV/B/BA | 6234 | S173P | T | C | O-linked site lost | 6 | 1 | Antigenic site V |
| RSV/B/BA | 6235 | S173F | C | T | O-linked site lost | 6 | 1 | Antigenic site V |
| RSV/B/BA | 6319 | N201S | A | G | N-linked site lost | 7 | 3 | Antigenic Site Øb |
| RSV/A/ON | 6039 | S105N | G | A | N-linked site gained | 5 | 1 | No |
| RSV/A/ON | 6089 | T122A | A | G | O-linked site lost | 2 | 1 | No |
| RSV/A/ON | 7277 | A518T | G | A | O-linked site gained | 2 | 1 | No |

**Supplementary Table 4)** A list of all non-synonymous variants found in the F gene.

| **Reference genome** | **Position** | **Amino acid substitution** | **Reference base** | **Variant**  **base** | **Potential change in glycosylation** | **Number of samples with this variant** | **Number of subjects with this variant** | **F antigenic site** |
| --- | --- | --- | --- | --- | --- | --- | --- | --- |
| RSV/B/BA | 5760 | L15F | C | T | No changes | 6 | 2 | No |
| RSV/B/BA | 5769 | N18H | A | C | N-linked site lost | 2 | 1 | No |
| RSV/B/BA | 5772 | A19T | G | A | O-linked site gained | 2 | 1 | No |
| RSV/B/BA | 5777 | L20F | G | T | No changes | 2 | 1 | No |
| RSV/B/BA | 5852 | L45F | G | T | No changes | 8 | 3 | Antigenic Site IIIa |
| RSV/B/BA | 6025 | A103V | C | T | No changes | 18 | 6 | No |
| RSV/B/BA | 6091 | L125P | T | C | No changes | 2 | 1 | No |
| RSV/B/BA | 6097 | V127A | T | C | No changes | 2 | 1 | No |
| RSV/B/BA | 6232 | L172Q | T | A | No changes | 18 | 6 | Antigenic Site V |
| RSV/B/BA | 6234 | S173P | T | C | O-linked site lost | 6 | 1 | Antigenic Site V |
| RSV/B/BA | 6235 | S173F | C | T | O-linked site lost | 6 | 1 | Antigenic Site V |
| RSV/B/BA | 6319 | N201S | A | G | N-linked site lost | 7 | 3 | Antigenic Site Ø*b* |
| RSV/B/BA | 6342 | Q209K | C | A | No changes | 9 | 4 | Antigenic Site Ø*b* |
| RSV/B/BA | 6700 | E328G | A | G | No changes | 6 | 2 | No |
| RSV/B/BA | 7122 | V469I | G | A | No changes | 3 | 1 | No |
| RSV/B/BA | 7442 | 575 | G | A | NA | 2 | 1 | No |
| RSV/A/ON | 5747 | T8S | A | T | No changes | 1 | 1 | No |
| RSV/A/ON | 5787 | C21Y | G | A | No changes | 1 | 1 | No |
| RSV/A/ON | 6039 | S105N | G | A | N-linked site gained | 5 | 1 | No |
| RSV/A/ON | 6089 | T122A | A | G | O-linked site lost | 2 | 1 | No |
| RSV/A/ON | 7277 | A518T | G | A | O-linked site gained | 2 | 1 | No |
